# Storing Data With Propargylic Amines Using Thin‐Layer Chromatography for the Data Retrieval

**DOI:** 10.1002/cplu.70202

**Published:** 2026-07-08

**Authors:** Miguel Mateus, Sundaravelu Nallappan, Lukas Rycek

**Affiliations:** ^1^ Department of Organic Chemistry Faculty of Science Charles University Prague Czech Republic

**Keywords:** A^3^ coupling, molecular data storing, multicomponent reaction, propargylic amines, silver catalysis

## Abstract

We developed a cost‐effective method for storing medium‐sized datasets in mixtures of propargylic amines as storage molecules. The synthesis of the molecules relies on a silver‐catalyzed multicomponent A^3^ coupling, enabling fast access to various propargylic amdines. The approach requires minimal synthetic effort, and simple TLC analysis ensures straightforward retrieval of the encoded data. Demonstrated on a QR code and a two‐bit image, this method enables long‐term preservation of static, small‐to‐medium‐sized datasets with high accuracy of recovery.

## Graphical abstract

Using our previously developed catalyst, we synthesized a series of propargylic amines and used mixtures of these compounds to encode two datasets: a QR code linking to our research group's website and an image of Charles IV, the founder of our university. The encoded data were subsequently recovered using TLC chromatography and decoded with in‐house developed software.

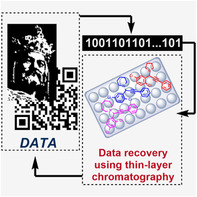

## 1 Introduction

The volume of data generated by humans is increasing at an unprecedented rate. In 2015, the total data volume was estimated at 15.5 zettabytes (ZB). By 2020, this figure had grown to 64 ZB, with projections suggesting it will reach 182 ZB by 2025, according to Statista [1]. Data has become indispensable in the modern world, encompassing a wide range of domains, including user‐generated content, enterprise and business data, scientific research, and more. Traditionally, data is stored on hard drives (HDs) or more advanced solid‐state drives (SSDs). These devices rely on the quantity of electrons within semiconductor components to store information, with data reading facilitated by the corresponding conductivity of the material. However, both storage capacity and data retention pose significant challenges for long‐term data preservation.

**FIGURE 1:**
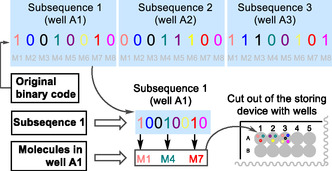
A principle of encoding binary information in the mixture of molecules.

The development of secure and long‐term data storage alternatives is critical and presents a significant technological challenge with far‐reaching implications for modern society. Molecular data storage offers a promising solution. Translating binary code into a linear macromolecule, whether natural or synthetic, was the initial step toward this approach [2, 3, 4, 5, 6, 7, 8, 9]. However, linear storage methods encounter limitations, such as strict control over the primary sequence, complex readout requirements, and the necessity to create unique polymeric molecules for each dataset. This poses a significant challenge for data encoding and readout. Additionally, DNA‐based storage is constrained by the use of only four monomers. These drawbacks can be addressed by using mixtures of small molecules to encode data (Figure 1). Here, binary code is divided into subsequences, with each position corresponding to a specific molecule (Figure 1; M1–M8). The presence of a molecule represents a binary “1,” while its absence signifies “0.” Examples include storing information in metabolomes [10], multicomponent Ugi products [11], or peptide mixtures [12], with matrix‐assisted laser desorption/ionization ‐ time of flight (MALDI‐TOF) analysis used for data retrieval. Alternatively, mixtures of fluorescent dyes printed on resin were analyzed via fluorescence microscopy [13]. Other approaches, such as using monomethoxypolyethylene glycols (mPEG) in combination with MALDI‐TOF for readout [14] or mixtures of random molecules and nuclear magnetic resonance (NMR) readout [15], were reported as well. Specific information (not necessarily a binary code) can be encoded in a mixture of isotopically labeled compounds, which provide mixture‐specific mass spectrometry (MS) fingerprints, based on the ratios of the isotopically labeled compounds [16, 17, 18]. The readout methods often rely on time‐consuming, expensive, or specialized equipment requiring analytical techniques. We recently developed a catalyst for the efficient preparation of propargylic amines via an A^3^ coupling [19, 20, 21]. A^3^ coupling is a multicomponent catalytic transformation, involving reaction of alkyne, aldehyde, and amine [22, 23]. This catalyst enabled us to synthesize a wide range of substrates, including those previously reported as challenging, such as aromatic aldehydes [24]. During our studies, we observed that several propargylic amines exhibited very distinct retention factors on thin‐layer chromatography (TLC). This observation led us to explore whether these synthesized propargylic amines could serve as the basis for an effective storage of data in the form of a binary code, with data retrieval facilitated by an inexpensive and straightforward TLC analysis.

## 2 Results and Discussion

### 2.1 Synthesis of Data Carriers

**SCHEME 1:**
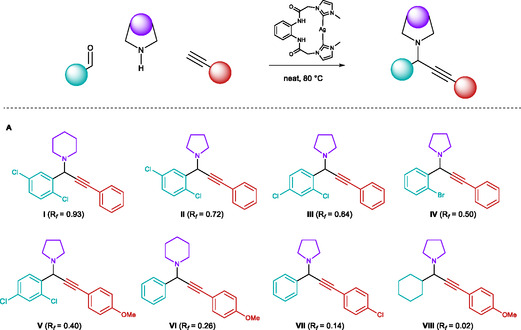
NHC–silver‐catalyzed A^3^ coupling reaction. Panel shows the eight propargylamines used for the QR code and the corresponding mean *R*_f_ values, under analytical conditions (see the Supporting Information for details).

In the initial stage of the investigation, we focused on the development of a TLC‐distinguishable collection of propargylic amines. Using our recently developed silver catalyst, we were allowed to quickly access a considerable library of propargylic amines. The multicomponent nature of the transformation gave us the opportunity to access such a library by means of different aldehydes, amines, and alkynes. The A^3^ coupling reaction allowed for fine‐tuning key parameters of the propargylic amines, such as retention factor and shape of their spots on TLC plates. While small, sharp‐edged spots were suitable, tailing, teardrop‐like spots were not suitable, as they spread over a large vertical distance during the TLC run, thereby reducing the number of compounds that could be effectively analyzed on the same TLC plate. Several trends were observed during the study. For example, employing pyrrolidine as the secondary amine resulted in higher polarity in the final products compared to those containing piperidine or azepine rings. Similarly, the 2,4‐dichloro substitution on the aromatic moiety derived from the aldehyde component led to higher polarity than the 2,5‐dichloro substitution. The polarity could be further fine‐tuned by altering the substitution patterns of the arylacetylenes. Notably, derivatives with 4‐methoxy or 4‐chloro substituents proved particularly effective. From over 30 compounds synthesized in the first series, eight were identified with distinct retention factor (*R*_f_) and suitable shapes of the spots on the TLC plates (Scheme 1A). Remarkably, many of the compounds identified originate from aromatic aldehydes, substrates that have, in some instances, proven challenging to access via A^3^ coupling. In contrast, our catalyst provides an efficient and reliable route to these valuable products.

### 2.2 Data Encoding, Analysis, and Readout

**FIGURE 2:**
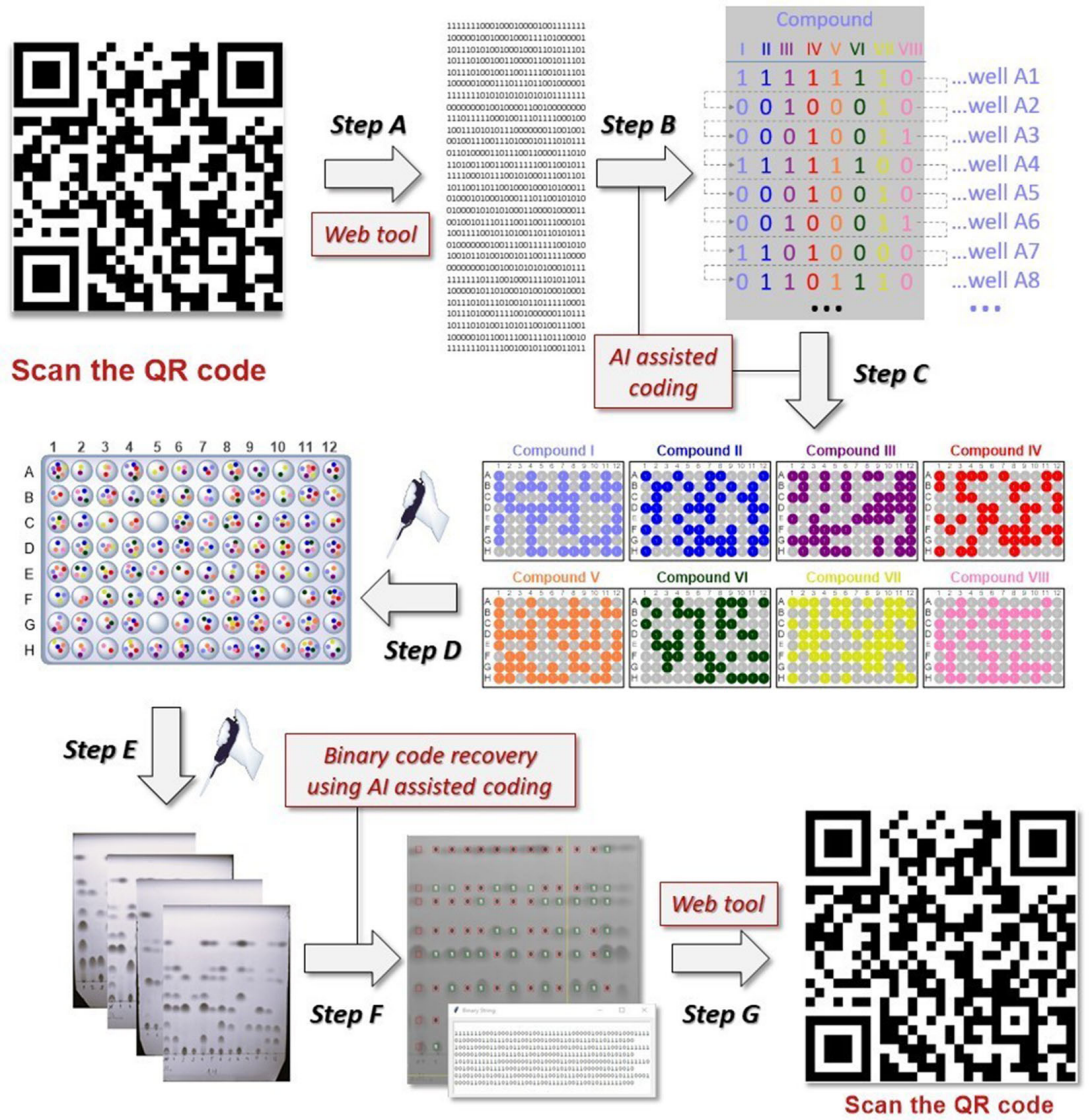
Encoding and decoding digital information using small molecules on TLC. Step A represents conversion of encoded data into a binary code. In step B, the binary code is inputted into a program (developed with AI assistance) and converted into a binary grid. The number of columns of the grid reflects the number of storing molecules. In step C, each vertical line of the binary grid (representing one storing molecule) is converted into a compound distribution map, visualizing the distribution of each compound within the storing device. Steps D and E represent distribution of molecules into storing plates and their analysis. Step F consists of converting the analytical data back to binary code, which is carried out by software developed in‐house with the assistance of AI. In the last step G, binary code is converted back to the data with 98.5% accuracy.

Having the eight suitable molecules in hand, we proceeded with the data encoding. To develop the entire process, we chose a QR code encoding the URL of our research group's webpage (Figure 2). In the first step, an online tool or custom software (developed with assistance from artificial intelligence) converts the QR code into binary code (step A). To visualize the molecular distribution within the model storage device (96‐well plate), we developed a Python script that arranges the binary code into a network with eight columns, corresponding to the eight storage molecules. In this arrangement, each horizontal line of the binary network represents one well of a 96‐well plate, while each vertical column corresponds to a specific compound (Figure 2, step B).

In this system, each well contains only those compounds represented by binary “1,” while molecules corresponding to binary “0” are absent. To visualize how the compounds are distributed across the entire storage plate (the compound distribution map), each vertical column (corresponding to a specific propargylic amine) is expanded into a 12‐column grid matching the layout of the 96‐well plate. In this way, a separate compound distribution map can be generated for each of the eight compounds used to encode the data. We developed a Python script, which, upon binary code input, can carry out the logical operation described above, and the output is the visualization of the molecular distribution across the storage device (Figure 2, step C—for the Python code and the real software output, see the Supporting Information). Having the distribution map of each molecule, methanolic solutions of the compounds were then dispensed into the storage plate using a 12‐channel pipette (step D). After the methanol evaporated, the plate was sealed and stored in a freezer, preserving the data securely.

For data recovery, the 96‐well plate is re‐dissolved in solvent, and TLC analysis is performed for each well (Figure 2, step E). Images of the TLC plates are captured and analyzed using custom software developed with assistance from artificial intelligence (Figure 2, steps D and E). The software provides a simple graphical user interface and processes images in two main steps: First, it identifies the expected locations of spots on the TLC plate based on their retention factors; second, it determines whether spots are present or absent by analyzing the *V* (value) component derived from the HSV (hue, saturation, value) color model, which represents image brightness or darkness. This information corresponds to the presence or absence of specific molecules in the mixture, enabling binary code retrieval. A detailed description of the software and its functionality is included in the supplementary materials. Using this process, we successfully recovered the QR code with 98.5% accuracy, enough for readability of the original data (QR codes incorporate built‐in error‐correction mechanisms, allowing them to remain readable even when approximately 1.5% of the recovered bits are incorrect).

**FIGURE 3:**
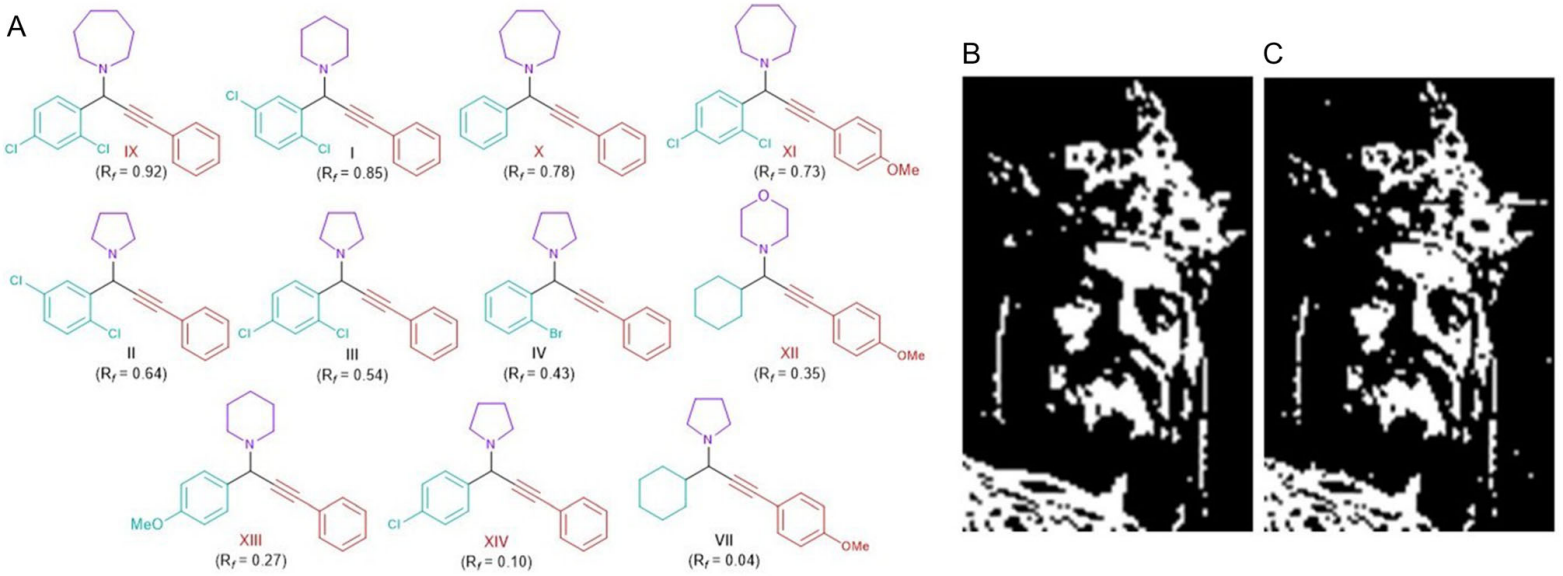
(A) New set of molecules, containing some molecules from previous set of molecules (numbered in black), and new molecules (numbered in red) with corresponding mean *R*_f_ values, under analytical conditions (see the Supporting Information for details). Visual comparison of the encoded input reference image (B) and recovered image from TLC‐based small‐molecule encoding (C), with 98.8% accuracy.

In the latter stage, we were able to extend the collection of amines to 11, which we used as the final set for the data encoding (Figure 3A). We replaced some of the original molecules, used for the eight‐molecular system, with different ones (numbered in red), which showed better shapes of the TLC spots (less tailing, sharp spots). With the updated set of molecules, we further demonstrate the utility of our method by encoding a larger dataset: a two‐bit depth black‐and‐white photograph of King Charles IV, the founder of our university. The original image (Figure 3B) was translated into binary code and encoded using the previously described protocol. We successfully encoded and decoded the image with an accuracy of 98.8% (Figure 3C, recovered image). In order to evaluate the potential for long‐term storage, we carried out the analysis after 6 months of storage. For storage purposes, the well plates containing the molecular mixtures were sealed with parafilm and stored under the air atmosphere in the freezer. After 6 months, the TLC analysis for data decoding was carried out. The analysis showed that the molecules remained stable under the applied conditions, with no evidence of decomposition observed, enabling complete recovery of the dataset.

### 2.3 Accuracy Improvement

**FIGURE 4:**
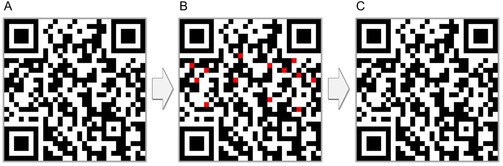
(A) Original QR code. (B) QR code recovered before fixing the errors (errors depicted in red). (C) QR code recovered after fixing protocol. Data recovered with 100% accuracy.

The reported 98.5% and 98.8% data recovery values lie close to the practical threshold limit of the method, with the remaining inaccuracy primarily arising from human error during data encoding as well as inherent limitations of the software. Human‐related errors can be effectively minimized by implementing a mandatory double‐check step immediately after encoding and before storing. We have developed a control software tool that compares the original binary code with the encoded binary output and identifies discrepancies between them (Code S4. The typical outcome of the software is shown in Figure S5). This allows for the detection of wells containing excess molecules as well as those missing components, which would lead to incorrect binary outputs. A key advantage of our approach is that such errors can be readily corrected by washing the affected wells and refilling them with the correct molecular mixture or simply adding the missing molecules. We applied this method to the originally encoded QR code (Figure 4A) and identified the 13 incorrect bits in 13 wells (Figure 4B). The positions were fixed, and after the second round of analysis, the binary code was recovered with 100% accuracy (Figure 4C).

## 3 Conclusion

In conclusion, we have developed a simple method for storing small‐sized datasets. We demonstrated its utility by encoding both the QR code for the URL of our group's website and a two‐bit depth image of Charles IV. This method is particularly suited for long‐term preservation of static, small‐to‐medium‐sized datasets. The presented TLC‐based molecular data storage platform provides a practical balance between storage capacity, simplicity, and instrumentation requirements. Its main advantage over previously reported approaches is the low cost of data readout, as the method does not require expensive instrumentation such as NMR or MS devices. The principal limitation of the method is the restricted number of molecules that can be reliably combined within a single mixture, since larger numbers of compounds may result in overlapping chromatographic spots or spots positioned too closely together, increasing the risk of misinterpretation during readout. Consequently, the storage density remains lower than that achievable using NMR‐ or MS‐based approaches. However, the presented methodology enables simultaneous analysis of multiple mixtures, which is not readily feasible with the abovementioned methods. In our standard workflow, 36 wells were analyzed simultaneously on a single TLC plate, corresponding to the recovery of 396 bits in a single analytical run. Moreover, a simple error‐checking and correction procedure for the stored molecules enables highly accurate data recovery, with recovery rates reaching up to 100%.

## Funding

This work was supported by the Univerzita Karlova v Praze (SVV 260690 and UNCE/SCI/014).

## Conflicts of Interest

The authors declare no conflicts of interest.

## Supporting information

The authors have cited additional references within the Supporting Information [25, 26, 27, 28, 29, 30, 31].

## Data Availability

The data that support the findings of this study are available in the supplementary material of this article.
