# Supplementary material for: Storing Data With Propargylic Amines Using Thin‐Layer Chromatography for the Data Retrieval
Source: Chempluschem. 2026 Jul 8;91(7):e70202. doi: 10.1002/cplu.70202 (PMC13345749; doi:10.1002/cplu.70202)
Supplement: Supplementary file 1 — The authors have cited additional references within the Supporting Information [25, 26, 27, 28, 29, 30, 31]. [file CPLU-91-e70202-s001.pdf]

Supplementary information for

Propargylic amines as data carriers with thin-layer chromatography for the data retrieval.

Miguel Mateus,[a] Sundaravelu Nallappan [a] and Lukas Rycek *[a]

Table of content

1. Experimental section…………………………………………………………………..2

2. Detailed process of the data encoding……..……………………………………..7

3. Detailed process of the data recovery……..……………………………………12

4. Used Python codes……………………………………………………………………22

5. Pictures of the TLC plates……………………………………………………….33

6. References…………………………………………………………………………..36

1. Experimental section

1.1. Materials and Physical Measurements

All chemicals were obtained from standard suppliers, including Sigma-Aldrich, Acros Organics, Alfa Aesar, Strem Chemicals, PENTA Chemicals, Fluorochem, and Cambridge Isotope Laboratories, Inc., and were used without further purification unless specified otherwise. Solvents employed in reactions were distilled and dried prior to use. Reaction progress was monitored by thin-layer chromatography (TLC) using Merck silica gel 60 F254 plates, with detection under a 254 nm UV lamp and visualization using Hanessian's stain (CAM).

NMR spectra were recorded on Bruker Avance III spectrometers (400 MHz and 600 MHz for ^{1}H NMR; 100 MHz and 150 MHz for ^{13}C NMR) and on a Varian NMR Solutions 300 (300 MHz for ^{1}H NMR; 75 MHz for ^{13}C NMR). Chemical shifts (δ) are reported in ppm relative to residual solvent signals. Mass spectrometry was conducted on a VG-Analytical ZAB SEQ instrument. Reactions requiring heating were performed using an oil bath as the heat source.

1.2. Synthesis and analytical data for applied compounds

General procedure for A3 coupling. A vial containing the catalyst (4.67 mg, 9.4 µmol, 0.5 mol%) was filled with an inert atmosphere, after which aldehyde 8 (1.5 mmol), amine 9 (1.5 mmol), and acetylene 10 (1.5 mmol) were added. The reaction mixture was stirred neat at 80 °C for 5 h. After completion, the mixture was dissolved in CH_2Cl_2 (20 mL) and washed with H_2O (3 × 20 mL). The combined aqueous layers were further extracted with CH_2Cl_2 (2 × 15 mL). The combined organic phases were dried over Na_2SO_4, filtered, and the solvent was removed under reduced pressure. Unless stated otherwise, the crude residue was purified by column chromatography on silica gel. The detailed experimental procedure and analytical data for the A^3 coupling are provided in the Supporting Information.

1-(1-(2,5-dichlorophenyl)-3-phenylprop-2-yn-1-yl)piperidine (I)

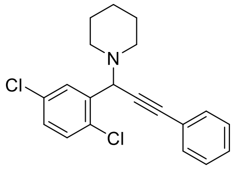

Following the general procedure: 2,5-dichlorobenzaldehyde (0.20 g, 1.7 mmol), piperidine (0.11 mL, 1.1 mmol), phenylacetylene (0.12 mL, 1.1 mmol) and catalyst (3 mol%) were used as starting substrates. After the work-up, the residue obtained was subjected to chromatographic column (Hexanes/EtOAc, 100/1) yielding 45% (0.18 g) of the desired compound as a yellow oil.

^{1}H NMR (400 MHz, $CDCl_3$) δ 7.72 (d, *J* = 2.6 Hz, 1H), 7.53 – 7.48 (m, 2H), 7.35 – 7.32 (m, 3H), 7.31 (s, 1H), 7.22 (dd, *J* = 8.5, 2.5 Hz, 1H), 5.03 (s, 1H), 2.59 (t, *J* = 5.5 Hz, 4H), 1.57 (tt, *J* = 11.1, 6.0 Hz, 4H), 1.44 (q, *J* = 5.7 Hz, 2H).

^{13}C NMR (101 MHz, $CDCl_3$) δ 138.43, 133.05, 132.36, 131.99, 130.99, 130.53, 128.94, 128.47, 122.99, 88.39, 84.92, 59.35, 50.93, 26.23, 24.52.

HRMS (ESI) m/z: Calcd for $C_{20}H_{20}Cl_2N^+$ = 344.0968; Found = 344.0965

1-(1-(2,5-dichlorophenyl)-3-phenylprop-2-yn-1-yl)pyrrolidine (II)

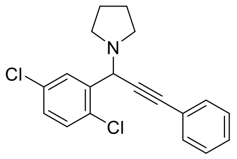

Following the general procedure: 2,5-dichlorobenzaldehyde (0.20 g, 1.1 mmol), pyrrolidine (0.09 mL, 1.1 mmol), phenylacetylene (0.12 mL, 1.1 mmol) and catalyst (3 mol%) were used as starting substrates After the work-up, the residue obtained was subjected to chromatographic column (Hexanes/EtOAc, 100/1) yielding 54% (0.20 g) of the desired compound as a yellow oil.

^{1}H NMR (400 MHz, $CDCl_3$) δ 7.91 (d, *J* = 2.5 Hz, 1H), 7.65 – 7.60 (m, 2H), 7.47 (dd, *J* = 4.1, 2.4 Hz, 3H), 7.45 (s, 1H), 7.36 (dd, *J* = 8.5, 2.6 Hz, 1H), 5.37 (s, 1H), 2.96 – 2.76 (m, 4H), 1.96 (ddd, *J* = 7.0, 4.7, 2.3 Hz, 4H).

^{13}C NMR (101 MHz, $CDCl_3$) δ 139.1, 132.7, 132.2, 131.9, 130.8, 130.1, 129.0, 128.5, 128.5, 122.9, 87.1, 85.6, 55.7, 50.5, 23.6.

HRMS (ESI) m/z: Calcd for $C_{19}H_{18}Cl_2N^+$ = 330.0811; Found = 330.0810

1-(1-(2,4-dichlorophenyl)-3-phenylprop-2-yn-1-yl)pyrrolidine (III)

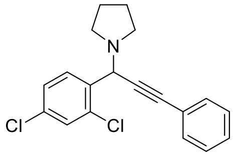

Following the general procedure: 2,4-dichlorobenzaldehyde (0.30 g, 1.7 mmol), pyrrolidine (0.15 mL, 1.7 mmol), phenylacetylene (0.19 mL, 1.7 mmol) and catalyst (3 mol%) were used as starting substrates. After the work-up, the residue obtained was subjected to chromatographic column (Hexanes/EtOAc, 100/1) yielding 46% (0.26 g) of the desired compound as a yellow oil.

^1H NMR (400 MHz, $CDCl_3$) δ 7.73 (d, J = 8.4 Hz, 1H), 7.48 – 7.44 (m, 2H), 7.40 (d, J = 2.1 Hz, 1H), 7.34 – 7.30 (m, 3H), 7.28 (d, J = 2.2 Hz, 1H), 7.25 (s, 1H), 5.23 (s, 1H), 2.78 – 2.63 (m, 4H), 1.85 – 1.76 (m, 4H).

^{13}C NMR (101 MHz, $CDCl_3$) δ 136.1, 134.6, 134.0, 131.9, 131.1, 129.5, 128.4, 127.3, 122.9, 86.9, 85.9, 55.3, 50.5, 23.6.

HRMS (ESI) m/z: Calcd for $C_{19}H_{18}Cl_2N^+$ = 330.0811; Found = 330.0808

The data is in accordance with the one reported in the literature.[1]

1-(1-(2-bromophenyl)-3-phenylprop-2-yn-1-yl)pyrrolidine (IV)

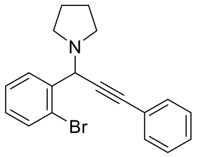

Following the general procedure: 2-bromobenzaldehyde (0.20 g, 1.1 mmol), pyrrolidine (0.09 mL, 1.1 mmol), phenylacetylene (0.12 mL, 1.1 mmol) and catalyst (3 mol%) were used as starting substrates After the work-up, the residue obtained was subjected to chromatographic column (Hexanes/EtOAc, 100/1) yielding 57% (0.21 g) the desired compound as a yellow oil.

^1H NMR (400 MHz, $CDCl_3$) δ 7.80 (dd, *J* = 7.7, 1.8 Hz, 1H), 7.58 (dd, *J* = 8.0, 1.3 Hz, 1H), 7.51 – 7.45 (m, 2H), 7.37 – 7.29 (m, 4H), 7.15 (td, *J* = 7.6, 1.7 Hz, 1H), 5.27 (s, 1H), 2.87 – 2.63 (m, 4H), 1.88 – 1.74 (m, 4H).

^{13}C NMR (101 MHz, $CDCl_3$) δ 139.0, 133.1, 131.9, 130.3, 129.1, 128.3, 128.2, 127.4, 124.5, 123.2, 86.8, 86.5, 58.3, 50.4, 23.6.

HRMS (ESI) m/z: Calcd for $C_{19}H_{19}BrN^+$ = 340.0696; Found = 340.0693

The data is in accordance with the one reported in the literature.[2]

1-(1-(2,4-dichlorophenyl)-3-(4-methoxyphenyl)prop-2-yn-1-yl)pyrrolidine (V)

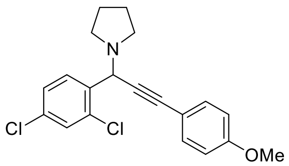

Following the general procedure: 2,4-dichlorobenzaldehyde (0.20 g, 1.1 mmol), pyrrolidine (0.09 mL, 1.1 mmol), 4-ethynilanisole (0.16 mL, 1.1 mmol) and catalyst (3 mol%) were used as starting substrates After the work-up, the residue obtained was subjected to chromatographic column (Hexanes/EtOAc, 100/1) yielding 50% (0.21 g) of the desired compound as a yellow oil.

^1H NMR (400 MHz, $CDCl_3$) δ 7.72 (d, *J* = 8.3 Hz, 1H), 7.44 – 7.35 (m, 3H), 7.26 (dd, *J* = 8.3, 2.2 Hz, 1H), 6.88 – 6.80 (m, 2H), 5.19 (s, 1H), 3.81 (s, 3H), 2.77 – 2.61 (m, 4H), 1.87 – 1.73 (m, 4H).

^{13}C NMR (101 MHz, $CDCl_3$) δ 159.7, 136.3, 134.6, 133.9, 133.3, 131.1, 129.5, 127.1, 115.1, 114.0, 86.7, 84.5, 55.4, 55.3, 50.5, 23.6.

HRMS (ESI) m/z: Calcd for $C_{20}H_{20}Cl_2NO^+$ = 360.0917; Found = 360.0917

1-(3-(4-methoxyphenyl)-1-phenylprop-2-yn-1-yl)piperidine (VI)

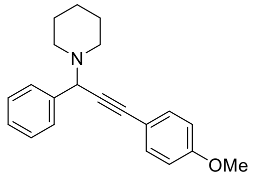

Following the general procedure: benzaldehyde (0.12 mL,1.2 mmol), piperidine (0.12 mL, 1.2 mmol), 4-ethynilanisole (0.15 mL, 1.2 mmol) and catalyst (0.5 mol%) were used as starting substrates, yielding 86% (0.31 g) of the desired compound as a yellow oil.

^{1}H NMR (400 MHz, $CDCl_3$) δ 7.66 (d, *J* = 6.8 Hz, 2H), 7.48 – 7.43 (m, 2H), 7.40 – 7.34 (m, 2H), 7.33 – 7.28 (m, 1H), 6.89 – 6.84 (m, 2H), 4.83 (s, 1H), 3.82 (s, 3H), 2.60 (s, 4H), 1.61 (dq, *J* = 12.9, 7.3, 6.1 Hz, 4H).

^{13}C NMR (101 MHz, $CDCl_3$) δ 159.6, 133.7, 133.3, 128.8, 128.2, 127.7, 114.1, 114.0, 88.0, 83.8, 62.5, 55.4, 50.8, 26.1, 24.4.

The data is in accordance with the one reported in the literature.[3]

1-(3-(4-chlorophenyl)-1-phenylprop-2-yn-1-yl)pyrrolidine (VII)

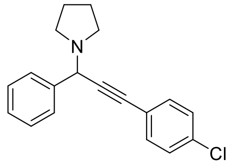

Following the general procedure, benzaldehyde (0.19 mL, 1.5 mmol), pyrrolidine (0.15 mL, 1.5 mmol), *p*-chlorophenylacetylene (205 mg, 1.5 mmol) and catalyst (0.5 mol%) were used as starting substrates. After the work-up, the residue obtained was subjected to chromatographic column (DCM/EtOAc, 100/1), yielding 71% (0.4 g) of the desired compound as a yellow oil.

^{1}H NMR (400 MHz, $CDCl_3$) δ 7.61 – 7.56 (m, 2H), 7.43 – 7.33 (m, 4H), 7.32 – 7.27 (m, 3H), 4.85 (s, 1H), 2.67 (td, *J* = 6.9, 5.7, 3.3 Hz, 4H), 1.88 – 1.74 (m, 4H).

^{13}C NMR (101 MHz, $CDCl_3$) δ 139.5, 134.2, 133.2, 128.7, 128.5, 128.4, 127.8, 121.8, 88.1, 85.8, 59.3, 50.5, 23.6.

The data is in accordance with the one reported in the literature.[4]

1-(1-cyclohexyl-3-(4-methoxyphenyl)prop-2-yn-1-yl)pyrrolidine (VIII)

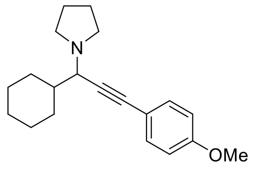

Following the general procedure: cyclohexanecarboxaldehyde (0.17 mL, 1.5 mmol), pyrrolidine (0.15 mL, 1.5 mmol), 4-ethynilanisole (0.20 mL, 1.5 mmol) and catalyst (0.5 mol%) were used as starting substrates, yielding 86% (0.38 g) of the desired compound as a pale-yellow oil.

^{1}H NMR (400 MHz, $CDCl_3$) δ 7.36 (d, 2H, *J* = 4.4 Hz), 6.82 (d, 2H, *J* = 7.8 Hz), 3.80 (s, 3H), 3.33 (d, 1H, *J* = 8.4 Hz), 2.73 (d, 2H, *J* = 6.6 Hz), 2.64 (d, 2H, *J* = 6.6 Hz), 2.08 (d, 1H, *J* = 12.6 Hz), 1.94 (d, 1H, *J* = 11.4 Hz), 1.78 (m, 6H), 1.67 (d, 1H, *J* = 12.0 Hz), 1.57 (m, 1H), 1.06 – 1.29 (m, 6H).

^{13}C NMR (151 MHz, $CDCl_3$) δ 159.3, 133.2, 116.0, 113.9, 86.3, 85.6, 61.5, 55.4, 50.2, 41.5, 30.9, 30.4, 26.8, 26.4, 23.7.

The data is in accordance with the one reported in the literature.[4]

1-(1-(2,4-dichlorophenyl)-3-phenylprop-2-yn-1-yl)azepane (IX)

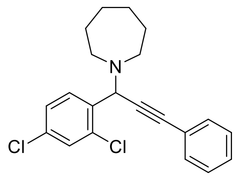

Following the general procedure: 2,4-dichlorobenzaldehyde (0.18 g, 1.0 mmol), azepane (0.11 ml, 1.0 mmol), and phenylacetylene (0.11 ml, 1.0 mmol), yielding 88% (0.31 g) of the desired compound as a yellow oil.

^{1}H NMR (400 MHz, $CDCl_3$) δ 7.71 (d, *J* = 8.3 Hz, 1H), 7.52 – 7.46 (m, 2H), 7.40 (d, *J* = 2.1 Hz, 1H), 7.33 (ddd, *J* = 4.5, 2.7, 1.4 Hz, 3H), 7.26 – 7.22 (m, 1H), 5.07 (s, 1H), 2.74 (ddd, *J* = 7.4, 4.8, 2.0 Hz, 4H), 1.69 – 1.43 (m, 8H).

^{13}C NMR (101 MHz, $CDCl_3$) δ 136.2, 135.7, 133.8, 131.9, 131.2, 129.7, 128.4, 128.4, 128.35, 126.5, 123.2, 87.5, 85.9, 59.3, 51.9, 31.0, 29.1, 27.2.

HRMS (ESI) m/z: Calcd for $C_{21}H_{22}Cl_2N^+$ = 358.1124; Found = 358.1130

1-(1,3-diphenylprop-2-yn-1-yl)azepane (X)

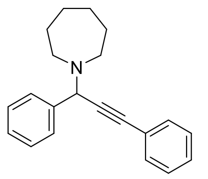

Following the general procedure: benzaldehyde (0.10 g, 0.9 mmol), azepane (0.11 ml, 0.9 mmol), and phenylacetylene (0.10 ml, 0.9 mmol), yielding 90% (0.25 g) of the desired compound as a transparent oil.

^{1}H NMR (400 MHz, $CDCl_3$) δ 7.75 – 7.70 (m, 2H), 7.56 – 7.50 (m, 2H), 7.39 – 7.28 (m, 6H), 4.95 (s, 1H), 2.76 (dd, *J* = 6.4, 4.0 Hz, 4H), 1.74 – 1.59 (m, 8H).

^{13}C NMR (101 MHz, $CDCl_3$) δ 131.9, 129.1, 128.4, 128.4, 128.1, 127.5, 123.5, 87.3, 86.8, 62.8, 52.8, 31.0, 29.0, 27.1.

The data is in accordance with the one reported in the literature.[5]

1-(1-(2,4-dichlorophenyl)-3-(4-methoxyphenyl)prop-2-yn-1-yl)azepane (XI)

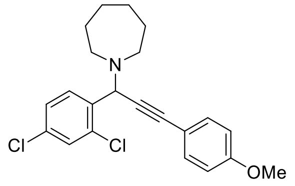

Following the general procedure: 2,4-dichlorobenzaldehyde (0.18 g, 1.0 mmol), azepane (0.11 ml, 1.0 mmol), and 4-ethynilanisole (0.13 g, 1.0 mmol), yielding 88% (0.34 g) of the desired compound as a yellow crystalline solid.

^{1}H NMR (400 MHz, $CDCl_3$) δ 7.71 (d, *J* = 8.3 Hz, 1H), 7.43 – 7.38 (m, 3H), 7.23 (dd, *J* = 8.3, 2.2 Hz, 1H), 6.87 – 6.84 (m, 2H), 5.05 (s, 1H), 3.82 (s, 3H), 2.73 (ddd, *J* = 7.5, 4.8, 2.3 Hz, 4H), 1.68 – 1.40 (m, 8H).

^{13}C NMR (101 MHz, $CDCl_3$) δ 159.68, 135.70, 133.77, 133.71, 133.31, 131.24, 129.70, 126.49, 115.28, 114.06, 87.34, 84.31, 59.28, 55.44, 51.88, 31.05, 29.11, 27.23.

HRMS (ESI) m/z: Calcd for $C_{22}H_{24}Cl_2NO^+$ = 388.1230; Found = 388.1229

4-(1-cyclohexyl-3-(4-methoxyphenyl)prop-2-yn-1-yl)morpholine (XII).

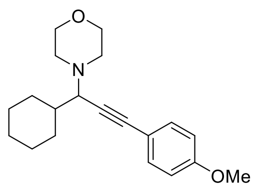

Following the general procedure: cyclohexanecarboxaldehyde (0.17 mL, 1.5 mmol), morpholine (0.13 mL, 1.5 mmol) 4-ethynilanisole (0.21 mL, 1.5 mmol) and catalyst (0.5 mol%) were used as starting substrates, yielding 94% (0.42 g) of the desired compound as a white crystalline substance.

^{1}H NMR (400 MHz, $CDCl_3$) δ 7.39 (d, *J* = 8.8 Hz, 2H), 6.84 (d, *J* = 8.8 Hz, 2H), 3.83 – 3.68 (m, 7H), 3.13 (d, *J* = 9.8 Hz, 1H), 2.79 – 2.65 (m, 2H), 2.57 – 2.46 (m, 2H), 2.17 – 1.99 (m, 2H), 1.83 – 1.55 (m, 4H), 1.30 – 0.93 (m, 5H).

^{13}C NMR (101 MHz, $CDCl_3$) δ 159.3, 133.1, 115.7, 113.9, 86.6, 85.1, 67.3, 64.1, 55.3, 50.0, 39.2, 31.1, 30.4, 26.8, 26.3, 26.1.

The data is in accordance with the one reported in the literature.[6]

1-(1-(4-methoxyphenyl)-3-phenylprop-2-yn-1-yl)piperidine (XIII)

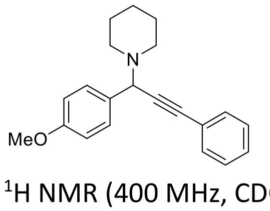

Following the general procedure: *p*-anisaldehyde (0.15 g, 1.1 mmol), piperidine (0.11 mL, 1.1 mmol), phenylacetylene (1.2 mL, 1.1 mmol) and catalyst (0.5 mol%) were used as starting substrates, yielding 65% (0.22 g) of the desired compound as a yellow oil.

^{1}H NMR (400 MHz, $CDCl_3$) δ 7.57 – 7.48 (m, 4H), 7.33 (td, *J* = 3.8, 1.9 Hz, 3H), 7.26 (d, *J* = 1.3 Hz, 1H), 6.93 – 6.86 (m, 2H), 4.75 (s, 1H), 3.82 (d, *J* = 1.4 Hz, 3H), 2.57 (q, *J* = 5.7 Hz, 4H), 1.59 (h, *J* = 6.8 Hz, 4H), 1.51 – 1.39 (m, 2H).

^{13}C NMR (101 MHz, $CDCl_3$) δ 159.1, 131.9, 129.8, 128.4, 128.1, 123.5, 113.5, 87.8, 86.5, 61.9, 55.4, 26.3, 24.6.

The data is in accordance with the one reported in the literature.[7]

1-(1-(4-chlorophenyl)-3-phenylprop-2-yn-1-yl)pyrrolidine (XIV)

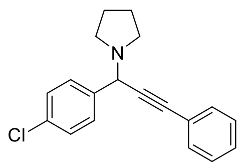

Following the general procedure: *p*-chlorobenzaldehyde (0.16 g, 1.1 mmol), pyrrolidine (0.09 mL, 1.1 mmol), phenylacetylene (1.2 mL, 1.1 mmol) and catalyst (0.5 mol%) were used as starting substrates, yielding 71% (0.24 g) of the desired compound as a yellow oil.

,^{1}H NMR (400 MHz, $CDCl_3$) δ 7.61 (d, 2H), 7.58 – 7.52 (d, *J* = 8.4 Hz, 2H), 7.46 – 7.29 (m, 5H), 4.95 (s, 1H), 2.76 (t, *J* = 7.0 Hz, 4H), 1.88 (t, *J* = 7.0 Hz, 4H).
^{13}C NMR (101 MHz, $CDCl_3$) δ 138.3, 133.4, 131.9, 129.7, 128.5, 128.4, 128.4, 123.1, 87.5, 86.1, 58.4, 50.2, 23.6.

The data is in accordance with the one reported in the literature.[8]

2. Detailed process of the data encoding

2.1) Step 1: Conversion of datasets to binary code.

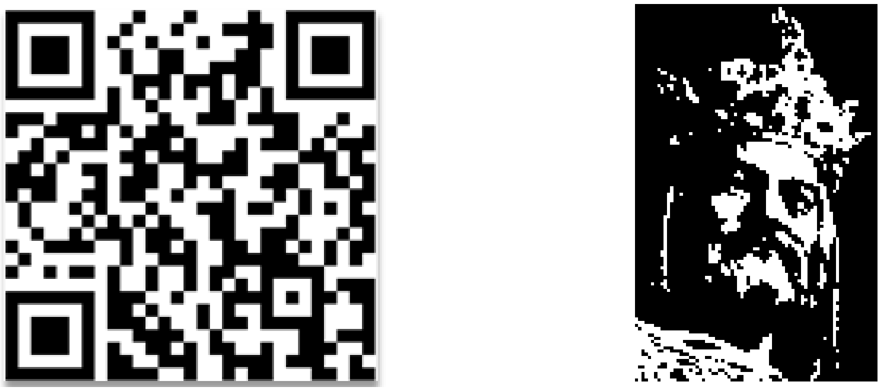

Figure S1. Encoded QR code and image of Charles IV.

The QR code (29 x 29 bits, 841 bits) and the image of king Charles IV (80 x 118 bits, 9440 bits, Figure S1) were converted to the binary codes, using the in house developed code (CODE 1), which converts dark pixels to "1" and light pixels to "0".

For the QR code, which was encoded by 8 molecules, the binary code was padded by 7 zeros, to make the length of the binary code dividable by 8. Similarly, binary code generated for the image of the king Charles IV was padded with 9 zeros, to make the overall length dividable by 11 (11 molecules used by storing).

Binary code of the QR code with padding:

1111111000100010000100111111111000001001000100011110100000110111010100100010001101011101101110100100110000110010
1110110111010010011001111001011101100000100011101110110010000011111111010101010101010111111100000000100100001100
1000000000111011111100010011101111000100100111010101110000000110010010010011100111010001011101011101101000011011100
1100001110101101001100110011111001100101111110001011100101000111001101101100110110010001000101000110100010100010
0011101100101010010000101010100011000010000110010010111011100110011100010110011110010110100110110101011010000000
1001110011111100101010010110100100101100111110000000000000100100101010100010111111111110111001000111101011011100000
0101101000101001000100011011101011101001011011111000110111010001111001000000110111101110101001101011001001110011
0000010110011100111101110010111111101111001001011000110110000000

Binary code for the image of the king Charles IV

00000000000000000000000000000000000000000000000000000000000000000000000000000000000000000000000000000000000000000000000000000010000
000000000000000000000000000000000000000000000000000000000000000000000011000000000000000000000000000000000000000000000000000 00000
0000000000000000000000001011000000000000000000000000000000000000000000000000000000000000000101110100000000000000000000000
00000000000000000000000000000000000000000000000000001100101100000000000000000000000000000000000000000000000000000000000000 00000
0000000000110000000000000000000000000000000000000000000000000000000000000000001011000000000000000000000000000000000000000000
000000000000000000000000000000000000001000011000000000000000000000000000000000000000000000000000000000000000000000000100 00000
0000000000000000000000000000000000000000000000000000000000000101001100000000000000000000000000000000000000000000000000000000000
000000000000000111111100000000000000000000000000000000000000000000000000000000000000000000110011100000000000000000000000 00000
0000000000000000000000000000000000000000000000001001111000000000000000000000000000000000000000000000000000000000000000000000001
10110000000000000000000000000000000000000000000000000000000000000000000001100011000000000000000000000000000000000000000000 00000
00000000000000000000000000001110000100000000000000000000000000000000000000000000000000000000000111110000000000111110111100000000000000
00000000000000000000000000000000000001111111111000000000111111111100000000000000000000000000000000000000000000000000000111111101 11101
1011100111100111000000000000000000000000000000000010000000000000000100011011101110011000110111100110000000000000000000000000000000000011
000000000000000011011000110001000011111111110000000000000000000000000000000000000010000000000000000111111011000010001111111111 11100
000000000000000000000000000000001100100000000000011111111000011011111111111011111000000000000000000000000000000000001100000000000 00
0011101101111111111111111111111100000000000000000000000000000000010011000010000000000000000011111111111001111111111111000000000000000 00000
00000000100100001100000000000000011111111111000011111111111000000000000000000000000000000000100000001100000000000000011111000100000 00011
1110011111000000000000000000000000000000000001110000000000000011100000110000101111111011111000000000000000000000000000000000000010 00111
000000000000110000000000011101100011111111000000000000000000000000000000000000000110000000000000000000000000000011111100001110111000 01111
000000000000000000000000000000000000000001000000000000001110110000111111110001111100000000000000000000000000000000000000000000 000000000
00001111111000011111111110110000000000000000000000000000000000000000000111000000000000000011111111000111111111110100000000000000000 00000
0000000000000000111001000111000001001111000001111111111011110000000000000000000000000000000000000000011000001111110000000 1111000011111
1111111110000000000000000000000000000000000000000000000001111100000000111110111111111111111100000000000000000000000000000000000000000 00000
1000001111000000011110111111000011111100000000000000000000000000000000000000000000000001001000111000111111000001 1111000000000000000
00000000000000000000000000100000000000000000000000111100111111000011111000000000000000000000000000000000100000000000000000000010011110 10011
011001111111111111001111100000000000000000000100000000000000000000000000000111111111100010011111111111111011100000000 00000000000110000000
000000000000000000000000111111111100111111111011110100000000000000000000000011100000000000000000000000011111111100001111000011111111 11111
000000000000000000000000000000000000000000100111111111111011111100010001111110000000000000000000000000000 0000000000000000011011111
1111111111111111111111100100110000000000000000000000000000000000000000000000001111111111111111111111011111000100000000000000000000000 00000
0000000000000000000000011111111111111111111100110000000000000000000000000000000000000000000000000000000000 0011111111111111111111111110010
000001100000000000000000000000000000000000000000000000000111111111111111111111100000000000100000000000000000000000000000000000000000 00000
000000001111111111111111111000100110000000000000000000000000000000000000000000000000000111111111 11111111100100111000000000000000
00000000000000000000000000000000001000000001111111111111111000000110000000000000000000000000000000000000000000000000011100000000111 11111
11111110000000100000000000000000000000000000000000000000000000000001000000011111110111111100000000 00000000000000000000000000000000000000000
00000000000000000000000111000000000111100000001000000000000000000000000000000000000000000000000000000000000111000000000000111000010 00000
000000000000000000000001000000000000000000000001000000000000001110000000100000110001000000000000000000000000001000000000000000000000001111000000
0111100000000111000110010000000000000000000000000000000011001000000000000011000111100000111100000000011100110000000000000000000000000000 00000
11000000000000111111111101100000111110000000001100110000000000000000000000000000000000001100000000000011111111111100000111110000000000010001
100000000000000000000000000000001000000000000011111111111000000111111000011000000110000000000000000000000000000000010000000000000 11111
1111000000011111100001100010011000000000000000000000000000000010000000000000011111111000000011111110000010110011000000000000000000000
00000000001000000000000000011111110000000011111110001001110011100000000000000000000000000000001100000000000000001111110000000000111 11110
01111110011100000000000000000000000000000100000000000000000000011100000000000000111111111111001110000000000000000000000000000000010000000000
000000001111100000000000000111111111110111100000000000000000000000000000000100000000000000000001111110000000000000000011111111100111100 00000
00000000000000000001000000000000000001111100000000000000000001111111101111100000000000000000000000000000100000000000000000001000000000000
000011100111111111111111000000000000000000000000000001000000000000000000000000000000000000001111000111111011111000000000000000000000000 00001
0000000000000000000000000000000010000111100011111011111010000000000000000000000000000000100000000000000000000000000000000000001111000111110011
11010000000000000000000000000001000000000000000010110000000000000000000000010111011000111001000000000000000000000000010000000000000010 11000
00000001100000000000111110000110010000000000000000000000000011000000000000001110000000000000000011000000011010000110010000000000000000
0000000011000000000000011110000000001100000001100000011000000010010000000000000000000000000001000000000000011110000000111110001111 00000
0011000000100100000000000000000000000000001000000000000001110000101111111101111100000011000000000100000000000000000000000000001000000000000
00000001001101111111111111110000000110000100001000000000000000000000000001100000000000000000001101100111111111111111000000110000100001000000
000000000000000000001000000000000000000111011111111111111110000011000010001000000000000000000000000000100000000000000000011011111111
1111111100101100001100010000000000000000000000000000000000000001100000000111011111111111110000110000110001000000000000000000000000 00001
00000000000001000000001110111011111111100011100001100010000000000000000000000010011000000000000000000000001110111000111111100011000110
0000000000000000000000000000001000000000000000000000000011110000000111111000010000100000000000000000000000000000001000000000000000 00000
0011100000000001111100000000000001100000000000000000000000000000000000000000000000001000000000011111000000000000011000000000000000000
000000000000000000000000000000000001000000000011111001001000000110000000000000000000000000000000000000000000000000000000000001 11101
00110100000001100000000000000000000000000000000000000000000000000000000100000001001100000100000000000000000000000000000000000000000000
000000000000000000000000000000000001000000110000000000000000000000000000000000000000000000000000000000000000000000000000000110 00000
00000000000000000000000000000000000000000000000000000000000000000000000010000000000000000000000000000000000000000000000000000000000000000
00000000000000000000000100000000000000000001000000000000000000000000000000000000000000000000000000110000000000000000011100 00000
000000000000000000000000000000000000000000000000000001000000000000000000011111000000000000000000000000000000000000000000000000000000000000
00100000000000000000010111111000000000000000000000000000000000000000000000000000000011000000000000000011111111111111111000000000 00000
000000000000000000000000000000001000000000000000000111100111111111111111110000000000000000000000010000000000000000000001000000000000000000
1111110011111111111111111100000000000000001110000000000000000010010000000000000000001111011111111110000000111111000000000000001100 00000
0000000000000100000000000000000011111100111111110000000001111100000000001111000000000000000000001000000000000000000111111111000111111100
0000000111110000000011110000000000000000000000000000000000000000011011110011000011111111111111111110011111100000000000000000000000010 00000
00000000001100111111111100011111111111111111111111110000000000000000000000000000000000000001111011111111111001110011111111111111111
1100000000000000000000000000000000000000111111111111000011011111010111111111111111100000000000000010000000000000000000000000011111 11100
1111000000111111111100011111111110000000000000001100000100000000000000000100111100011111111101111111111110000011111111000000000000000100
000100000000000000001111111111110001101101101100111110000111111111000000000000100000000000000000000000110111011111000011110011100 01101
11111111111111110000000000110000000000000000000000001101110111100000111111111111101100111111110001000000000011000000000000000000000000000
01001110111110000111101111111110000111110111000110000000100000000000000000000000000000000000

2.2) Step 2: Conversion of binary codes into compound distribution maps.

The generated binary codes were used as inputs to Code 2, which converts it into the distribution of each molecule in the storing 96 well plate. The program takes a single long binary ***mother string*** and splits it into multiple ***daughter strings***, one for each compound. This is done by cycling through the mother string so that each daughter string gets every nth bit (where n is the number of compounds). Each daughter string is then divided into rows of 12 bits to create a grid representation, allowing a visual distribution of 1s and 0s. Essentially, the mother string's bits are evenly distributed among the compounds, preserving their order, and displayed in a structured, grid-like format for easier analysis. The real outcomes of the software are shown below (Figure S2):

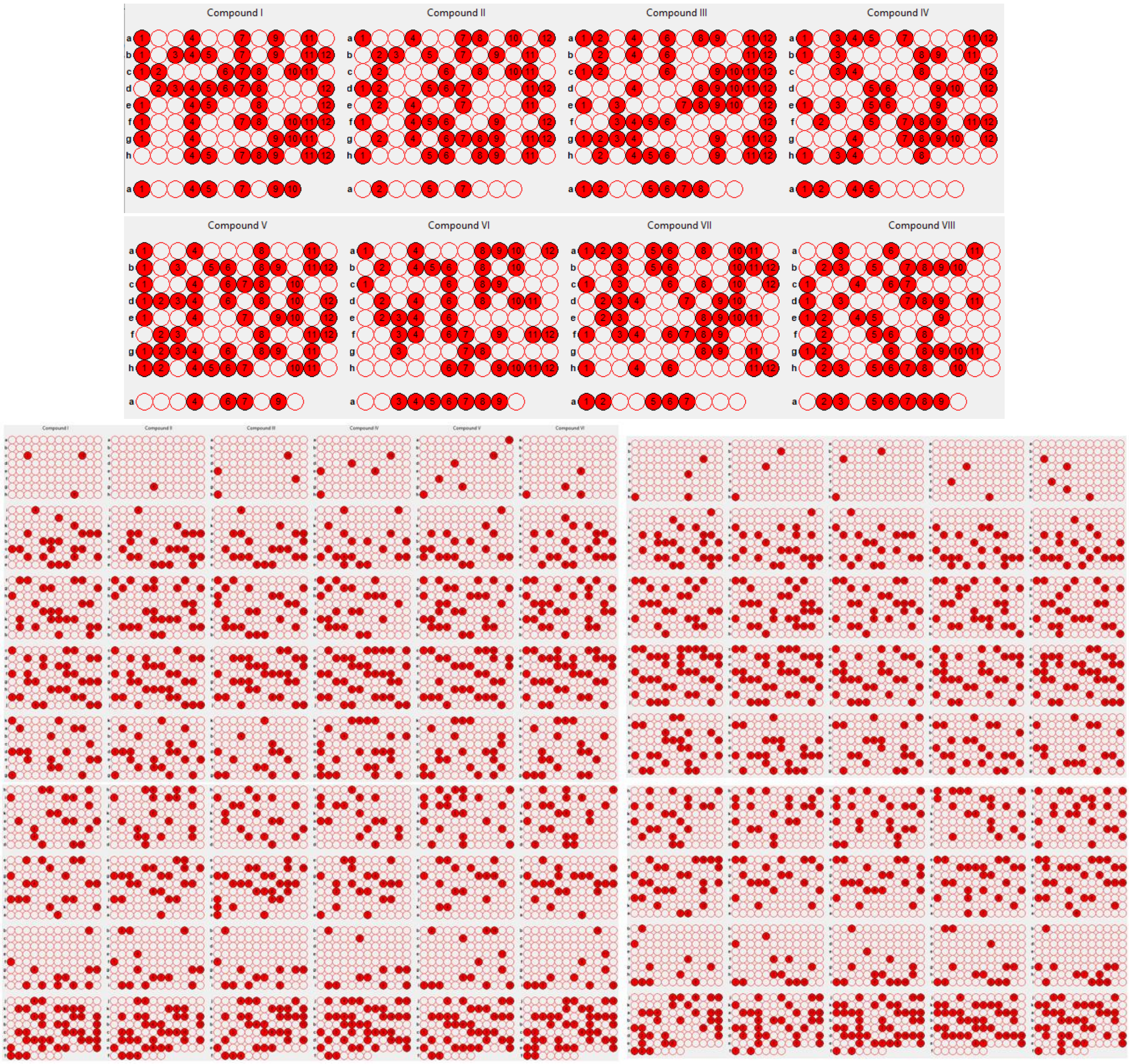

Figure S2. Compound distribution maps for the encoded QR code (above), and for the image of Chrles IV (below).

2.3) Step 3: Streamlining the distribution of molecules into the storing plate.

Once the molecular distribution pattern for each compound is generated by the software (Figure S3A), the compounds are transferred using a three–well-plate system and a 12-channel pipette to ensure accuracy and efficiency.

Well plate 1 (Figure S3B) is used to hold pipette tips. The arrangement of the tips mirrors the molecular distribution pattern provided by the software, allowing direct correspondence between tip position and destination wells.

Well plate 2 (Figure S3C) contains the methanolic stock solutions of the coding compounds, transferred from the prepared stock solutions (1 mg of compound per 0.5 mL of the solvent). Each row corresponds to a different compound (e.g., row 1 contains compound I, row 2 contains compound II, and so on).

Well plate 3 (Figure S3D) serves as the final storage plate, where the compounds are transferred according to the predefined distribution pattern.

The 12-channel pipette (50–300 µL) is fitted with tips from row 1 of well plate 1. Using these tips, 50 µL of solution of compound I is aspirated from the stock solution plate (well plate 2) and dispensed into the corresponding wells of row 1 in the storage plate (well plate 3), following the distribution pattern. The tips are then discarded, and the pipette is fitted with tips from row 2 of well plate 1. Compound I is then continued by transferring 50 µL from row 2 of the stock solution plate (well plate 2) into row 2 of the storage plate (well plate 3). This process is repeated sequentially for all rows of compound I. Once compound I has been fully transferred, the same procedure is applied to compounds II, III, and so on until all compounds are processed according to the predefined pattern. (Figure 3S shows the transfer of 8 compounds, in latter stage, 11 compounds were used). After all the compounds are transferred, the methanol is left to evaporate in the fume hood overnight.

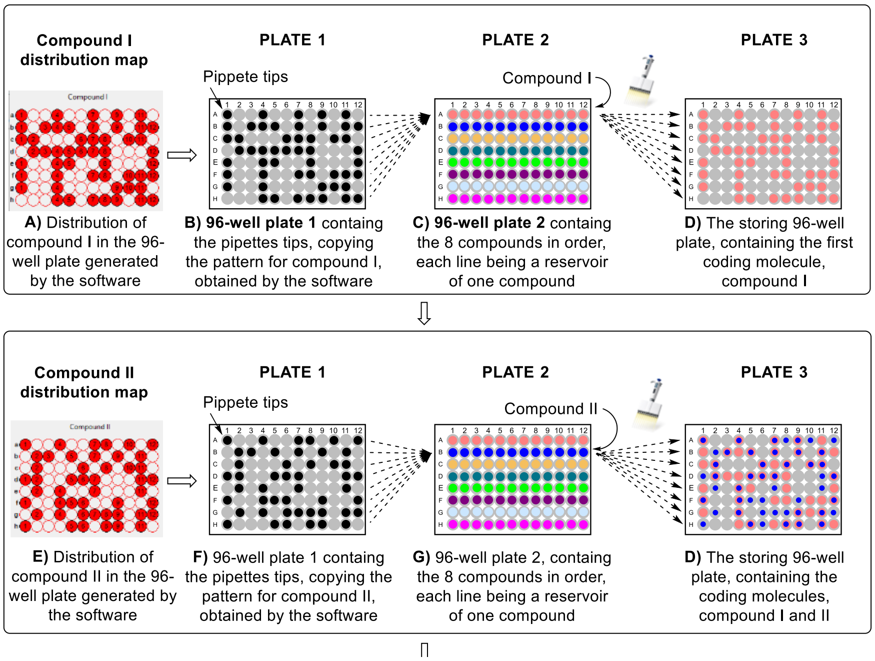

Figure S3. Schematic depiction of the encoding process.

3. Detailed process of the data recovery

In this section, the step-by-step process for binary code recovery is described. For the analysis, the stored mixtures in the 96-well plates were re-dissolved in methanol (each well fully filled with methanol). The 20 x 20 TLC plates (Merck silica gel 60 F254 plates) were cut in half, and samples were applied along the long edge of the plate. Samples were applied to the TLC plate using the provided 10 µL microcapillaries (Hirschmann ringcaps®). Each sample was taken once as a single aliquot and spotted directly onto the plate, with no repeated sampling from the same well. Three rows of wells, (36 wells in total), corresponding to 288 bits in case of 8-compound system, and 396 bits in case of 11-compound system, were spotted on a single TLC plate. Prior to development, the elution chamber (22 x 22 x 6.5 cm) was charged with 200 mL of the solvent mixture (for 8 molecule system: hexanes and ethyl acetate, 10:1; for 11 molecules system: hexanes and ethyl acetate, 20:1). Each plate was developed three consecutive times under identical conditions, with drying between each elution step. Rf values were determined from multiple independent TLC plates. For each plate, individual spot Rf values were measured and used to calculate the plate mean and within-plate standard deviation (SD). Overall Rf values were obtained by averaging the mean Rf values from all plates, while reproducibility between plates was evaluated as the standard deviation of plate means (between-plate SD). For the 8-compound system, all nine TLC plates containing coding mixtures were included in the analysis. For the 11-molecule system, nine TLC plates were randomly selected and used for the statistical evaluation. Plates without detectable spots were excluded from the analysis.

Compound 8 system:

Compound VIII
Plate 1: values = [0.021, 0.021, 0.019], mean = 0,020, SD = 0,001, n = 3
Plate 2: values = [0.019, 0.019, 0.024, 0.022, 0.031, 0.039, 0.027], mean = 0,026, SD = 0,007, n = 7
Plate 3: values = [0.048, 0.029, 0.031, 0.024, 0.045, 0.036, 0.036, 0.033], mean = 0,033, SD = 0,008, n = 8
Plate 4: no spots
Plate 5: values = [0.023, 0.023, 0.023, 0.021], mean = 0,023, SD = 0,001, n = 4
Plate 6: values = [0.046, 0.016, 0.023, 0.039, 0.021], mean = 0,029, SD = 0,011, n = 5
Plate 7: values = [0.018, 0.017, 0.014, 0.021, 0.021, 0.018, 0.018], mean = 0,018, SD = 0,002, n = 7
Plate 8: values = [0.019, 0.019, 0.024, 0.021, 0.019, 0.027, 0.021], mean = 0,022, SD = 0,003, n = 7
Plate 9: values = [0.011, 0.015, 0.015, 0.015, 0.019, 0.019, 0.032], mean = 0,018, SD = 0,007, n = 7

FINAL RESULT: Rf = 0,024 ± 0,003 (within-plate SD), reproducibility = ± 0,005 (between-plate SD), n = 8 plates (1 empty excluded)

Compound VII:
Plate 1: values = [0.147, 0.154, 0.159, 0.162, 0.157, 0.150, 0.150, 0.150], mean = 0,154, SD = 0,005, n = 8
Plate 2: values = [0.145, 0.145, 0.143, 0.145, 0.138, 0.128], mean = 0,141, SD = 0,006, n = 6
Plate 3: values = [0.182, 0.163, 0.151, 0.165, 0.153, 0.148], mean = 0,160, SD = 0,013, n = 6
Plate 4: values = [0.143, 0.141, 0.141, 0.141, 0.146, 0.133], mean = 0,141, SD = 0,005, n = 6
Plate 5: values = [0.140, 0.113, 0.122, 0.131, 0.133, 0.131, 0.129], mean = 0,129, SD = 0,008, n = 7
Plate 6: values = [0.132, 0.146, 0.153, 0.144, 0.141, 0.132], mean = 0,141, SD = 0,008, n = 6
Plate 7: values = [0.128, 0.123, 0.117], mean = 0,123, SD = 0,006, n = 3
Plate 8: values = [0.109, 0.119, 0.111, 0.101, 0.098], mean = 0,108, SD = 0,009, n = 5
Plate 9: values = [0.099, 0.095, 0.102, 0.099, 0.110], mean = 0,101, SD = 0,006, n = 5

FINAL RESULT: Rf = 0,135 ± 0,009 (within-plate SD), reproducibility = ± 0,020 (between-plate SD), n = 9 plates

Compound VI:
Plate 1: values = [0.29, 0.285, 0.287, 0.285, 0.285, 0.283], mean = 0,286, SD = 0,002, n = 6
Plate 2: values = [0.283, 0.275, 0.28, 0.273, 0.273, 0.273], mean = 0,276, SD = 0,004, n = 6

Plate 3: values = [0.321, 0.282, 0.294, 0.282], mean = 0,295, SD = 0,018, n = 4
Plate 4: values = [0.281, 0.274, 0.266, 0.266, 0.264, 0.261], mean = 0,269, SD = 0,008, n = 6
Plate 5: values = [0.248, 0.264, 0.292, 0.297, 0.285, 0.29, 0.257], mean = 0,276, SD = 0,018, n = 7
Plate 6: values = [0.269, 0.285, 0.289, 0.278], mean = 0,280, SD = 0,009, n = 4
Plate 7: values = [0.284, 0.248, 0.255], mean = 0,262, SD = 0,020, n = 3
Plate 8: values = [0.239, 0.223, 0.218, 0.223, 0.215, 0.215], mean = 0,222, SD = 0,009, n = 6
Plate 9: values = [0.229, 0.227, 0.238, 0.227, 0.253, 0.235, 0.253], mean = 0,238, SD = 0,012, n = 7

FINAL RESULT: Rf = 0,261 ± 0,010 (within-plate SD), reproducibility = ± 0,024 (between-plate SD), n = 9 plates

Compound V
Plate 1: values = [0.435, 0.428, 0.425, 0.42], mean = 0,427, SD = 0,007, n = 4
Plate 2: values = [0.406, 0.406, 0.413, 0.413, 0.425, 0.406, 0.396, 0.386], mean = 0,406, SD = 0,011, n = 8
Plate 3: values = [0.457, 0.39, 0.4, 0.407, 0.414, 0.409], mean = 0,413, SD = 0,022, n = 6
Plate 4: values = [0.405, 0.417, 0.412, 0.407, 0.407, 0.402, 0.394, 0.412], mean = 0,407, SD = 0,006, n = 8
Plate 5: values = [0.377, 0.37, 0.368, 0.409, 0.384], mean = 0,382, SD = 0,016, n = 5
Plate 6: values = [0.433, 0.426, 0.414, 0.4, 0.419, 0.417], mean = 0,418, SD = 0,011, n = 6
Plate 7: values = [0.394, 0.384, 0.394, 0.396, 0.386, 0.395, 0.385, 0.374], mean = 0,388, SD = 0,008, n = 8
Plate 8: values = [0.371, 0.379, 0.385, 0.363, 0.377, 0.361, 0.369, 0.353], mean = 0,370, SD = 0,011, n = 8
Plate 9: values = [0.343, 0.348, 0.356, 0.356], mean = 0,351, SD = 0,006, n = 4

FINAL RESULT: Rf = 0,396 ± 0,012 (within-plate SD), reproducibility = ± 0,026 (between-plate SD), n = 9 plates

Compound IV
Plate 1: values = [0.53, 0.518, 0.527, 0.53, 0.52, 0.525, 0.52], mean = 0,524, SD = 0,005, n = 7
Plate 2: values = [0.507, 0.51, 0.502, 0.498, 0.495], mean = 0,502, SD = 0,006, n = 5
Plate 3: values = [0.524, 0.502, 0.514, 0.5], mean = 0,510, SD = 0,011, n = 4
Plate 4: values = [0.51, 0.513, 0.513, 0.505, 0.53], mean = 0,514, SD = 0,010, n = 5
Plate 5: values = [0.474, 0.471, 0.494, 0.48, 0.474, 0.513, 0.494], mean = 0,486, SD = 0,015, n = 7
Plate 6: values = [0.546, 0.532, 0.521, 0.512], mean = 0,528, SD = 0,015, n = 4
Plate 7: values = [0.495, 0.487, 0.484, 0.489, 0.481, 0.476], mean = 0,486, SD = 0,007, n = 6
Plate 8: values = [0.477, 0.467, 0.477, 0.477], mean = 0,475, SD = 0,005, n = 4
Plate 9: values = [0.443, 0.445, 0.447, 0.464], mean = 0,450, SD = 0,010, n = 4

FINAL RESULT: Rf = 0,495 ± 0,012 (within-plate SD), reproducibility = ± 0,026 (between-plate SD), n = 9 plates

Compound III
Plate 1: values = [0.675, 0.667, 0.665, 0.667, 0.66, 0.672, 0.658, 0.658], mean = 0,666, SD = 0,006, n = 8
Plate 2: values = [0.657, 0.645, 0.645, 0.643, 0.623], mean = 0,643, SD = 0,011, n = 5
Plate 3: values = [0.684, 0.675, 0.653, 0.651, 0.651, 0.644, 0.644], mean = 0,658, SD = 0,015, n = 7
Plate 4: values = [0.656, 0.658, 0.663, 0.668, 0.676, 0.678], mean = 0,667, SD = 0,008, n = 6
Plate 5: values = [0.639, 0.628, 0.630, 0.637, 0.625, 0.630], mean = 0,632, SD = 0,005, n = 6
Plate 6: values = [0.706, 0.690, 0.681, 0.676, 0.667, 0.676, 0.694], mean = 0,684, SD = 0,014, n = 7
Plate 7: values = [0.656, 0.651, 0.653, 0.648, 0.642, 0.636, 0.639], mean = 0,646, SD = 0,007, n = 7
Plate 8: values = [0.631, 0.631, 0.634, 0.629, 0.621, 0.615, 0.610], mean = 0,625, SD = 0,009, n = 7
Plate 9: values = [0.600, 0.603, 0.609, 0.611, 0.616, 0.622], mean = 0,610, SD = 0,009, n = 6

FINAL RESULT: Rf = 0,644 ± 0,009 (within-plate SD), reproducibility = ± 0,020 (between-plate SD), n = 9 plates

Compound II
Plate 1: values = [0.748, 0.736, 0.734, 0.734, 0.727, 0.727], mean = 0,734, SD = 0,009, n = 6
Plate 2: values = [0.720, 0.713, 0.710, 0.698, 0.700, 0.700], mean = 0,707, SD = 0,009, n = 6
Plate 3: values = [0.742, 0.722, 0.722, 0.720, 0.715], mean = 0,724, SD = 0,010, n = 5

Plate 4: values = [0.731, 0.731, 0.729, 0.739, 0.736, 0.749, 0.751], mean = 0,738, SD = 0,009, n = 7
Plate 5: values = [0.720, 0.715, 0.710, 0.710, 0.706, 0.710], mean = 0,712, SD = 0,005, n = 6
Plate 6: values = [0.757, 0.752, 0.741, 0.762], mean = 0,753, SD = 0,009, n = 4
Plate 7: values = [0.722, 0.719, 0.716, 0.721, 0.724, 0.723, 0.715, 0.715], mean = 0,720, SD = 0,004, n = 8
Plate 8: values = [0.708, 0.703, 0.700, 0.706, 0.708, 0.703], mean = 0,705, SD = 0,003, n = 6
Plate 9: values = [0.683, 0.693, 0.700], mean = 0,692, SD = 0,009, n = 3

FINAL RESULT: Rf = 0,720 ± 0,006 (within-plate SD), reproducibility = ± 0,014 (between-plate SD), n = 9 plates

Compound I
Plate 1: values = [0.941, 0.938, 0.936, 0.933, 0.931], mean = 0,936, SD = 0,004, n = 5
Plate 2: values = [0.930, 0.925, 0.925, 0.920, 0.918, 0.913, 0.908, 0.906], mean = 0,918, SD = 0,008, n = 8
Plate 3: values = [0.947, 0.940, 0.928, 0.926, 0.923, 0.926, 0.921], mean = 0,930, SD = 0,009, n = 7
Plate 4: values = [0.950, 0.947, 0.947, 0.942, 0.945, 0.950, 0.952, 0.957], mean = 0,949, SD = 0,005, n = 8
Plate 5: values = [0.949, 0.945, 0.945, 0.943, 0.945, 0.947, 0.945], mean = 0,945, SD = 0,002, n = 7
Plate 6: values = [0.975, 0.970, 0.968, 0.972, 0.984], mean = 0,974, SD = 0,006, n = 5
Plate 7: values = [0.954, 0.943, 0.937, 0.926], mean = 0,940, SD = 0,012, n = 4
Plate 8: values = [0.926, 0.923, 0.926, 0.926, 0.926, 0.926, 0.926], mean = 0,926, SD = 0,001, n = 7
Plate 9: values = [0.916, 0.927, 0.929, 0.933, 0.935, 0.942], mean = 0,930, SD = 0,008, n = 6

FINAL RESULT: Rf = 0,928 ± 0,008 (within-plate SD), reproducibility = ± 0,018 (between-plate SD), n = 9 plates

11 compound system:

Compound 11
Plate 1: values = [0.035, 0.031, 0.041, 0.038], mean = 0,036, SD = 0,004, n = 4
Plate 2: values = [0.040, 0.034, 0.035, 0.032, 0.036, 0.040, 0.045, 0.033, 0.044, 0.031, 0.031, 0.036], mean = 0,036, SD = 0,005, n = 12
Plate 3: values = [0.028, 0.035, 0.027, 0.037, 0.033], mean = 0,032, SD = 0,005, n = 5
Plate 4: values = [0.039, 0.039, 0.038, 0.038, 0.033, 0.040, 0.036, 0.036, 0.043, 0.039], mean = 0,038, SD = 0,003, n = 10
Plate 5: values = [0.040, 0.045, 0.034, 0.035, 0.032, 0.032], mean = 0,036, SD = 0,005, n = 6
Plate 6: values = [0.034, 0.033, 0.039], mean = 0,035, SD = 0,003, n = 3
Plate 7: values = [0.039, 0.037, 0.039, 0.034, 0.040], mean = 0,038, SD = 0,002, n = 5
Plate 8: values = [0.041, 0.039], mean = 0,040, SD = 0,001, n = 2
Plate 9: values = [0.036, 0.045], mean = 0,041, SD = 0,006, n = 2

FINAL RESULT: Rf = 0,037 ± 0,004 (within-plate SD), reproducibility = ± 0,003 (between-plate SD), n = 9 plates

Compound 10
Plate 1: values = [0.093, 0.093, 0.084, 0.081, 0.099, 0.096], mean = 0,091, SD = 0,007, n = 6
Plate 2: values = [0.099, 0.089, 0.090, 0.091, 0.094, 0.095, 0.085, 0.093, 0.087, 0.095, 0.089, 0.086], mean = 0,091, SD = 0,004, n = 12
Plate 3: values = [0.091, 0.098, 0.093, 0.088, 0.102], mean = 0,094, SD = 0,005, n = 5
Plate 4: values = [0.093, 0.096, 0.096, 0.095, 0.106, 0.094, 0.102, 0.105, 0.105], mean = 0,099, SD = 0,005, n = 9
Plate 5: values = [0.102, 0.099, 0.103, 0.097, 0.093, 0.094], mean = 0,098, SD = 0,004, n = 6
Plate 6: values = [0.103, 0.101, 0.094, 0.095], mean = 0,098, SD = 0,005, n = 4
Plate 7: values = [0.106, 0.095, 0.086, 0.088], mean = 0,094, SD = 0,010, n = 4
Plate 8: values = [0.090], mean = 0,090, SD = 0,000, n = 1
Plate 9: values = [0.102, 0.099, 0.098, 0.103, 0.104], mean = 0,101, SD = 0,003, n = 5

FINAL RESULT: Rf = 0,095 ± 0,005 (within-plate SD), reproducibility = ± 0,004 (between-plate SD), n = 9 plates

Compound 9
Plate 1: values = [0.250, 0.251, 0.257, 0.253, 0.250], mean = 0,252, SD = 0,003, n = 5
Plate 2: values = [0.264, 0.252, 0.242, 0.246, 0.251, 0.241, 0.264, 0.252, 0.245, 0.242, 0.251, 0.245], mean = 0,250, SD = 0,008, n = 12
Plate 3: values = [0.268, 0.271, 0.271], mean = 0,270, SD = 0,002, n = 3
Plate 4: values = [0.279, 0.279, 0.278, 0.277, 0.291], mean = 0,281, SD = 0,006, n = 5
Plate 5: values = [0.294, 0.302, 0.298, 0.299, 0.289, 0.289, 0.289, 0.279], mean = 0,292, SD = 0,007, n = 8
Plate 6: values = [0.267, 0.268, 0.279, 0.270], mean = 0,271, SD = 0,005, n = 4
Plate 7: values = [0.267, 0.262, 0.248], mean = 0,259, SD = 0,010, n = 3
Plate 8: values = [0.266], mean = 0,266, SD = 0,000, n = 1
Plate 9: values = [0.263, 0.271, 0.271, 0.284], mean = 0,272, SD = 0,009, n = 4

FINAL RESULT: Rf = 0,269 ± 0,006 (within-plate SD), reproducibility = ± 0,014 (between-plate SD), n = 9 plates

Compound 8
Plate 1: values = [0.324, 0.324, 0.327, 0.320, 0.322, 0.327], mean = 0,324, SD = 0,003, n = 6
Plate 2: values = [0.330, 0.320, 0.318, 0.311, 0.308, 0.316, 0.317, 0.418, 0.422, 0.422, 0.326, 0.316], mean = 0,344, SD = 0,049, n = 12
Plate 3: values = [0.353, 0.341, 0.348], mean = 0,347, SD = 0,006, n = 3
Plate 4: values = [0.372, 0.371, 0.355, 0.370, 0.376], mean = 0,369, SD = 0,008, n = 5
Plate 5: values = [0.378, 0.382, 0.382, 0.372, 0.380, 0.373], mean = 0,378, SD = 0,005, n = 6
Plate 6: values = [0.343, 0.351, 0.360, 0.368, 0.368, 0.372], mean = 0,360, SD = 0,011, n = 6
Plate 7: values = [0.327, 0.332], mean = 0,330, SD = 0,004, n = 2
Plate 8: values = [0.350, 0.346], mean = 0,348, SD = 0,003, n = 2
Plate 9: values = [0.333, 0.352, 0.361, 0.362, 0.358], mean = 0,353, SD = 0,012, n = 5

FINAL RESULT: Rf = 0,350 ± 0,011 (within-plate SD), reproducibility = ± 0,018 (between-plate SD), n = 9 plates

Compound 7
Plate 1: values = [0.412, 0.419, 0.412, 0.410, 0.407], mean = 0,412, SD = 0,004, n = 5
Plate 2: values = [0.414, 0.402, 0.403, 0.391, 0.392, 0.389, 0.394], mean = 0,398, SD = 0,009, n = 7
Plate 3: values = [0.435, 0.434, 0.429, 0.433], mean = 0,433, SD = 0,003, n = 4
Plate 4: values = [0.446, 0.449, 0.449, 0.441, 0.444, 0.444, 0.443, 0.461], mean = 0,447, SD = 0,006, n = 8
Plate 5: values = [0.465, 0.465, 0.472, 0.465, 0.459], mean = 0,465, SD = 0,005, n = 5
Plate 6: values = [0.439, 0.440, 0.440, 0.447, 0.447], mean = 0,443, SD = 0,004, n = 5
Plate 7: values = [0.409, 0.409, 0.406], mean = 0,408, SD = 0,002, n = 3
Plate 8: values = [0.446, 0.430, 0.436], mean = 0,437, SD = 0,008, n = 3
Plate 9: values = [0.420, 0.415, 0.426, 0.435, 0.436, 0.443], mean = 0,429, SD = 0,011, n = 6

FINAL RESULT: Rf = 0,428 ± 0,006 (within-plate SD), reproducibility = ± 0,021 (between-plate SD), n = 9 plates

Compound 6
Plate 1: values = [0.527, 0.530, 0.520, 0.526, 0.527], mean = 0,526, SD = 0,004, n = 5
Plate 2: values = [0.539, 0.516, 0.524, 0.501, 0.498, 0.499, 0.500, 0.509], mean = 0,511, SD = 0,015, n = 8
Plate 3: values = [0.543, 0.538, 0.545], mean = 0,542, SD = 0,004, n = 3
Plate 4: values = [0.565, 0.564, 0.577], mean = 0,569, SD = 0,007, n = 3
Plate 5: values = [0.574, 0.570, 0.578, 0.578, 0.575, 0.565], mean = 0,573, SD = 0,005, n = 6
Plate 6: values = [0.565, 0.566, 0.566, 0.567, 0.577, 0.577], mean = 0,570, SD = 0,006, n = 6
Plate 7: values = [0.510, 0.511], mean = 0,510, SD = 0,001, n = 2
Plate 8: values = [0.540, 0.547], mean = 0,544, SD = 0,005, n = 2
Plate 9: values = [0.533, 0.527, 0.549], mean = 0,536, SD = 0,011, n = 3

FINAL RESULT: Rf = 0,539 ± 0,006 (within-plate SD), reproducibility = ± 0,024 (between-plate SD), n = 9 plates

Compound 5
Plate 1: values = [0.635, 0.631, 0.637, 0.634], mean = 0,634, SD = 0,003, n = 4
Plate 2: values = [0.645, 0.628, 0.621, 0.626, 0.619, 0.594, 0.594, 0.610], mean = 0,617, SD = 0,018, n = 8
Plate 3: values = [0.642, 0.654], mean = 0,648, SD = 0,008, n = 2
Plate 4: values = [0.659, 0.658, 0.653, 0.668], mean = 0,660, SD = 0,006, n = 4
Plate 5: values = [0.679, 0.679, 0.675, 0.676, 0.677, 0.667], mean = 0,676, SD = 0,004, n = 6
Plate 6: values = [0.678, 0.669, 0.669, 0.680, 0.674], mean = 0,674, SD = 0,005, n = 5
Plate 7: values = [0.619, 0.616], mean = 0,618, SD = 0,002, n = 2
Plate 8: values = [0.640, 0.643], mean = 0,642, SD = 0,002, n = 2
Plate 9: values = [0.630, 0.629, 0.649], mean = 0,636, SD = 0,011, n = 3

FINAL RESULT: Rf = 0,644 ± 0,007 (within-plate SD), reproducibility = ± 0,022 (between-plate SD), n = 9 plates

Compound 4
Plate 1: values = [0.727, 0.740, 0.737], mean = 0,735, SD = 0,007, n = 3
Plate 2: values = [0.744, 0.738, 0.731, 0.725, 0.722, 0.696, 0.700, 0.702], mean = 0,720, SD = 0,019, n = 8
Plate 3: values = [0.719, 0.731, 0.742], mean = 0,731, SD = 0,012, n = 3
Plate 4: values = [0.744, 0.747, 0.746], mean = 0,746, SD = 0,002, n = 3
Plate 5: values = [0.751, 0.748, 0.752, 0.760, 0.757], mean = 0,754, SD = 0,005, n = 5
Plate 6: values = [0.764, 0.765, 0.765, 0.766, 0.776, 0.777], mean = 0,769, SD = 0,006, n = 6
Plate 7: values = [0.709, 0.705, 0.706, 0.712], mean = 0,708, SD = 0,003, n = 4
Plate 8: values = [0.724, 0.716], mean = 0,720, SD = 0,006, n = 2
Plate 9: values = [0.717, 0.703, 0.715], mean = 0,712, SD = 0,008, n = 3

FINAL RESULT: Rf = 0,732 ± 0,008 (within-plate SD), reproducibility = ± 0,021 (between-plate SD), n = 9 plates

Compound 3
Plate 1: values = [0.781, 0.781, 0.783, 0.789], mean = 0,784, SD = 0,004, n = 4
Plate 2: values = [0.799, 0.779, 0.769, 0.773, 0.744, 0.745, 0.742], mean = 0,764, SD = 0,021, n = 7
Plate 3: values = [0.776, 0.775, 0.773, 0.773, 0.782, 0.781], mean = 0,777, SD = 0,004, n = 6
Plate 4: values = [0.790, 0.789, 0.788, 0.792, 0.803, 0.802], mean = 0,794, SD = 0,007, n = 6
Plate 5: values = [0.802, 0.806, 0.806, 0.814, 0.815], mean = 0,809, SD = 0,006, n = 5
Plate 6: values = [0.822, 0.823, 0.817, 0.827, 0.829], mean = 0,824, SD = 0,005, n = 5
Plate 7: values = [0.757, 0.756, 0.753, 0.751, 0.757], mean = 0,755, SD = 0,003, n = 5
Plate 8: values = [0.762], mean = 0,762, SD = 0,000, n = 1
Plate 9: values = [0.757, 0.767], mean = 0,762, SD = 0,007, n = 2

FINAL RESULT: Rf = 0,782 ± 0,007 (within-plate SD), reproducibility = ± 0,025 (between-plate SD), n = 9 plates

Compound 2
Plate 1: values = [0.850, 0.862, 0.868, 0.866], mean = 0,862, SD = 0,008, n = 4
Plate 2: values = [0.858, 0.845, 0.846, 0.817, 0.807, 0.819], mean = 0,832, SD = 0,021, n = 6
Plate 3: values = [0.827, 0.842, 0.842, 0.843, 0.847], mean = 0,840, SD = 0,008, n = 5
Plate 4: values = [0.853, 0.852, 0.852, 0.850, 0.858, 0.865, 0.864], mean = 0,856, SD = 0,006, n = 7
Plate 5: values = [0.860, 0.867, 0.864, 0.875, 0.866], mean = 0,866, SD = 0,005, n = 5
Plate 6: values = [0.880, 0.881, 0.892, 0.890], mean = 0,886, SD = 0,006, n = 4
Plate 7: values = [0.832, 0.827, 0.818, 0.832], mean = 0,827, SD = 0,007, n = 4
Plate 8: values = [0.819], mean = 0,819, SD = 0,000, n = 1
Plate 9: values = [0.811, 0.841, 0.844], mean = 0,832, SD = 0,018, n = 3

FINAL RESULT: Rf = 0,848 ± 0,009 (within-plate SD), reproducibility = ± 0,022 (between-plate SD), n = 9 plates

Compound 1
Plate 1: values = [0.931, 0.947, 0.941, 0.950], mean = 0,942, SD = 0,008, n = 4
Plate 2: values = [0.924, 0.915, 0.905, 0.889, 0.884, 0.889], mean = 0,901, SD = 0,016, n = 6
Plate 3: values = [0.901, 0.904, 0.904, 0.903, 0.905, 0.905], mean = 0,904, SD = 0,002, n = 6
Plate 4: values = [0.922, 0.926, 0.910, 0.920], mean = 0,920, SD = 0,007, n = 4
Plate 5: values = [0.921, 0.929, 0.941, 0.934, 0.934, 0.931], mean = 0,932, SD = 0,007, n = 6
Plate 6: values = [0.946, 0.943, 0.938], mean = 0,942, SD = 0,004, n = 3
Plate 7: values = [0.896, 0.898, 0.885, 0.884], mean = 0,891, SD = 0,007, n = 4
Plate 8: values = [0.895, 0.911], mean = 0,903, SD = 0,011, n = 2
Plate 9: values = [0.878, 0.906], mean = 0,892, SD = 0,020, n = 2

FINAL RESULT: Rf = 0,915 ± 0,009 (within-plate SD), reproducibility = ± 0,020 (between-plate SD), n = 9 plates

After development of the TLC, a photograph of the TLC plate was taken and uploaded into our in-house software (Code 3). This software, developed with the assistance of artificial intelligence, performs the decoding. The decoding process is illustrated and described in Figure S4.

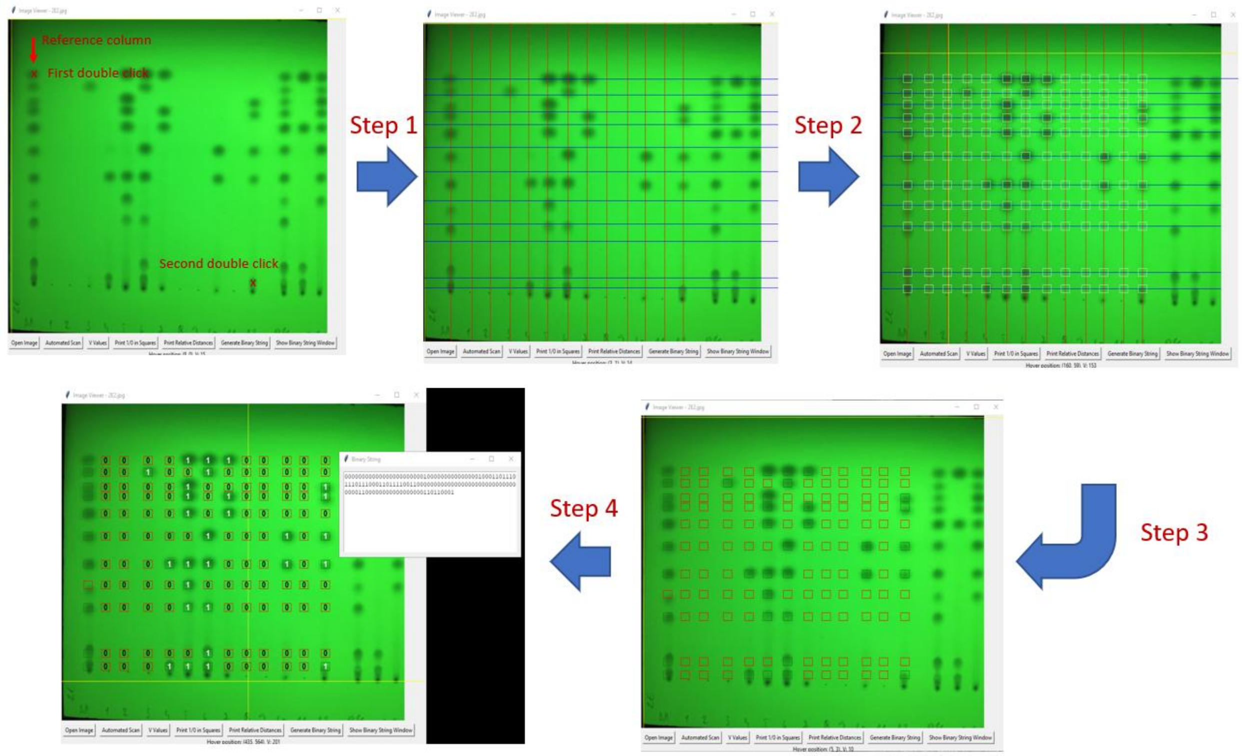

Figure S4. The overall process of the data recovery.

Step 1. Image upload and definition of frontier points of the grid
The image of the TLC plate (taken by a camera or scanned by Translum) is uploaded to the software. The image contains the reference column (mixture of all the compounds) on the far-left side and 12 columns for the analysis. Each column in the image corresponds to one of twelve wells in one horizontal line of the

96-well plate. In the step 1 a grid is generated. This is achieved by a double click on the top spot of the reference column (defining left top frontier of the grid) and on the position corresponding to the lowest spot of the 12th column (defining bottom right frontier of the grid). The grid generates automatically. Horizontal lines of the grid are generated based on the expected retention factors of the molecules, which are defined in the software. Vertical lines position is defined based on the equal distances between each of the columns inputted in the software.

Step 2. Grid adjustment

Due to the relatively long development times associated with each analytical run, slight variations in retention factors may occur between TLC plates. These inconsistencies can lead to misalignment of the predefined analytical grid. To address this, grid adjustment is performed either automatically or manually. Automated grid correction is carried out using the “Automated Scan” function, which analyzes the intensity profiles (specifically, the value component V in the HSV color space) in the vicinity of the original horizontal and vertical grid lines. If a region of higher darkness is detected near an existing line, the line is repositioned accordingly to better align with the observed spot pattern.

Manual grid correction is also supported, allowing the user to refine the line positions by direct interaction (e.g., dragging).

Following grid correction, rectangular regions are defined at each intersection point of the horizontal and vertical lines. These regions represent the expected locations of TLC spots. The average darkness (V value) within each region is calculated, and based on a predefined threshold, each region is assigned a binary value: 1 if a spot is detected (i.e., sufficiently dark), or 0 if no spot is present.

Step 3. Detection of the spots.

To determine the presence of TLC spots, the average V value (value component from the HSV color space, representing brightness) within each rectangular grid region is compared to the average V value of the surrounding background area located between the horizontal grid lines.

A threshold criterion is applied: if the average V value within a rectangle is less than or equal to 90% of the background V value, the region is classified as containing a spot. This 90% threshold was empirically determined to provide optimal accuracy in spot detection. If the brightness inside the rectangle is comparable to that of the surrounding area, the region is classified as not containing a spot.

For visualization purposes, rectangles identified as containing a spot are highlighted in green, while those without spots are marked in red. Manual correction of spot assignments is supported and can be performed by clicking the middle mouse button on the respective rectangle.

Step 4. Binary string generation.

In the final stage of the analysis, a binary code is generated based on the presence or absence of spots within the defined grid regions. Rectangles marked in red—indicating the absence of a spot—are assigned a binary value of 0, reflecting the absence of the corresponding compound in the storage mixture. Conversely, green rectangles—where a spot is detected—are assigned a binary value of 1.

The resulting binary sequence is displayed in a pop-up window. Additional images can be uploaded and analyzed using the same procedure. The binary strings generated from

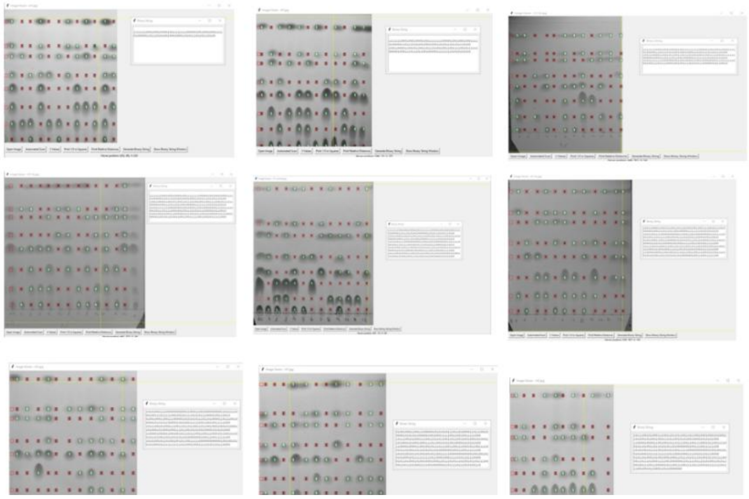

Figure S5. Gradual generation of the recovered binary code.

subsequent analyses are appended to the previously generated sequences, allowing for cumulative data collection across multiple TLC plates (Figure S5). The recovered code was in the end compared to the input binary code, to determine the efficiency of the recovery, using home-developed software (Code 4). The same code, allowed us to identify the problematic wells, that could be repaired before the storing (Figure S6)

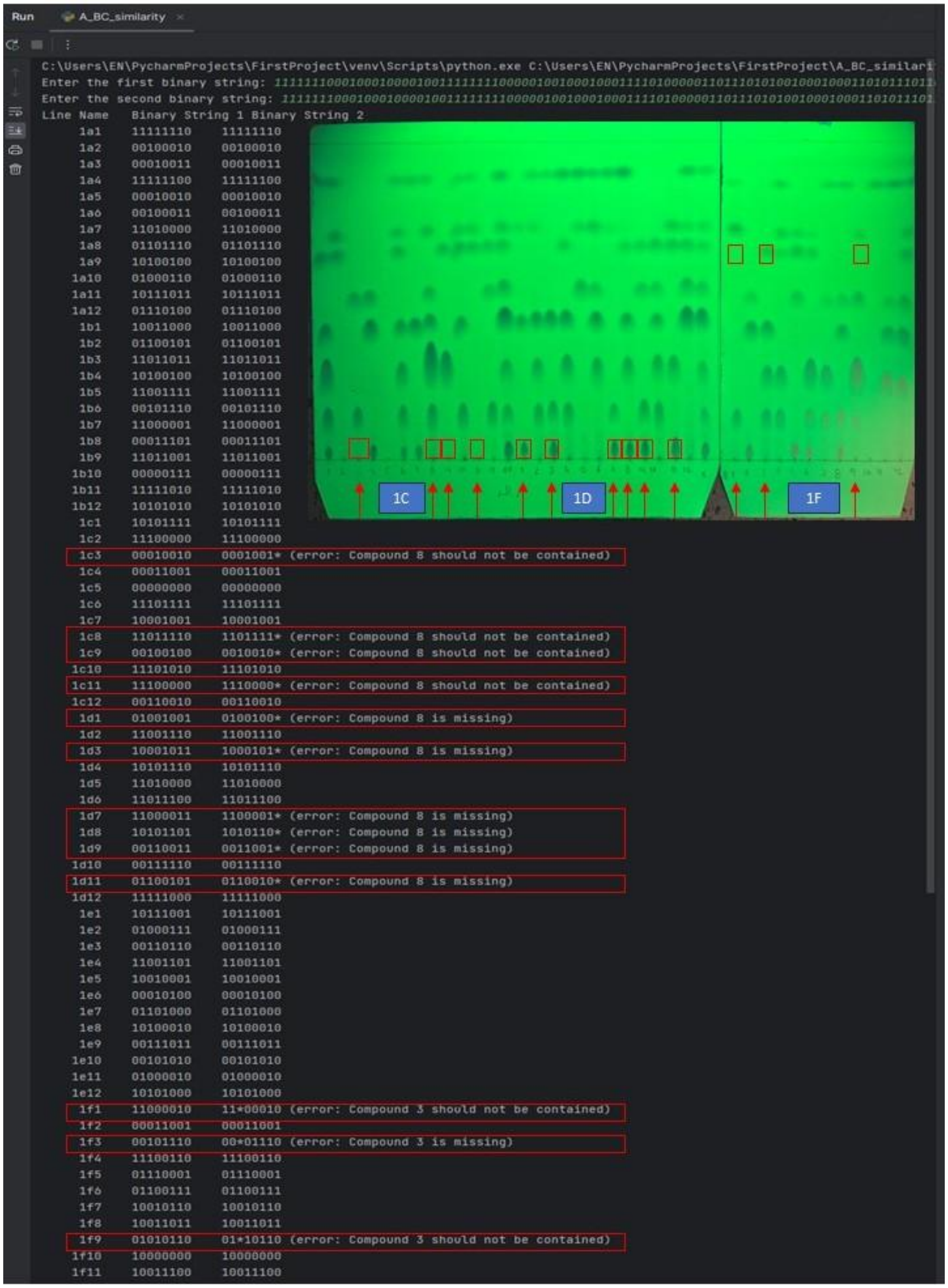

Figure S6. Comparing the codes and identification of the problematic positions.

Recovered binary code of the QR code with padding (errors depicted in red):

1111111000100010000100111111111000001001000100011110100000110111010100100010001101011101101110100100110000110010 1
11011011101001001100111100101110110000010001110111011001000001111111101010101010101010111111110000000001001100011001
00000000111011111100010011101111100100101111010101011100001001100100100100011001110100010101010111011010000110111 00
1100001010101100001100100011111001100100111110001011100101000111001101101100110110010001000101000110100010100010
00111011001010100100001010101000111000100001100100001110111001100111000101100111100101101001101101110110100000000
10011100111111001010100101101001001011001111100000000000001001001010101000101111111111011100100011110101101110000
01011010001010010001000110111010111010010110111110001101110100011110010000001101111011101010011010110010011 10011
00000101100111001111011100101111111011110010010110001101100000000

Recovered binary code of the QR code, after reparatory session (with padding):

1111111000100010000100111111111000001001000100011110100000110111010100100010001101011101101110100100110000110010 1
11011011101001001100111100101110110000010001110111011001000001111111101010101010101010111111110000000001001000011001
0000000011101111110001001110111100010010011101010101110000000110010010010011100111010001011101010111011010000110111 00
1100001110101101001100110011111001100101111110001011100101000111001101101100110110010001000101000110100010100010
00111011001010100100001010101000110000100001100100101110111001100111000101100111100101101001101101010110100000000
10011100111111001010100101101001001011001111100000000000001001001010101000101111111111011100100011110101101110000
01011010001010010001000110111010111010010110111110001101110100011110010000001101111011101010011010110010011 10011
00000101100111001111011100101111111011110010010110001101100000000

Recovered binary code for the image of the king Charles IV with padding (errors in red):

000000000000000000000000000000000000000000000000000000000000000000000000000000000000000000000000000000000000000000000010000
00000000000000000000000000000000000000000000000000000000000000000000000110000000000000000000000000000000000000000000000000
00000000000000000000000110000000000000000000000000000000000000000000000000000000000000000000000100110100000000000000000000
00000000000000000000000000000000000000000000000011001010000000000000000000000000000000000000000000010000000000000000000000
00000000000000000000000000000000000000000000000000000000000000000000000000000000101100000000000000000000000000000000000
000000000000000000000000000000000100001100000000000000000000000000000000000000000000000000000000000000000000000000010000000
00000000000000000000000000000000000000000000000000000000000010000110000000000000000000000000000000000000000000000000000
00000000000000011111110000000000000000000000000000000000000000000000000000000000000000001100111000000000000000000000000
00000000000000000000000000000000000000000000011110000000000000000000000000000000000000000000000000000000000000000000001
10110000000000000000000000000000000000000000000000000000000000000011000110000000000000000000000000000000000000000000
00000000000000000000000001010000100000000000000000000000000000000000000000000000011111000000000011110111100000000000
00000000000000000000000000000000001111111111000000001111111110000000000000000000000000000000000000000000001011000111101
10111001110001110000000000000000000000000000100000000000000100011011101110111000110111100110000000000000000000000000011
00000000000000011011000110001000011111111100000000000000000000000000001000000000000000111111011000010001111111111111100
000000000000000000000000000011000000000000011111110000110111111111110111110000000000000000000000000000001000000000000000
0011101100011111011100111110110000000000000000000001000100001000000000000000111111111000111111101111100000000000000000
00000000100100001100000000000000011111111111000011111111111000000000000000000000001000000011000000001111100010000000011
11100111110000000000000000000000000000000000011100000000000111000001100001011111110111110000000000000000000000001000111
000000000001100000000001110110001111111000000000000000000000000000000011000000000000000000000000011111000111011100011111
00000000000000000000000000000000000010000000000000111011000011111111000111100000000000000000000000000000000000000000000
00000000000000011111100001111111111011000000000000000000000111000000000000000111111110001111111111010000000000000000000
00000000000000011100100011100000100111100000111111111011110000000000000000000000000000001100001111110000000111100011111
11111110000000000000000000000000000000000000000000111110000000111101111111111111000000000000000000000000000000000000000
100000111100000001111011110000111111000000000000000000000000000000000000000000010010001110001111100000111110000000000000
00000000000000000000000010000000000000000011110011111100001111000000000000000000000010000000000000000000001001111010011
0110011111111111110011110000000000000000001000000000000000000001111111111001001111111111111011100000000000000110000000
000000000000000000000001111111111001111111011101000000000000000000110000000000000000000001111111110000111100001111111111
00000000000000000000000000000000000000001001111111111111011111100010001111110000000000000000000000000000000000110011111
111110111111011101110010110000000000000000000000000000000000000011111111111111111111011110001000000000000000000000000
000000000000000000000111111111111111111110011000000000000000000000000000000000000000000011111111111111111111111111110010
00000110000000000000000000000000000000000000000000111111111111111111110000000000010000000000000000000000000000000000000
00000000111111111111111110001001100000000000000000000000000000000000000000000000000111001111111111110010011110000000000000
00000000000000000000000000000100000000111111111011111000001110000000000000000000000000000000000000000000011100000111111 1
11111110000000100000000000000000000000000000000000000000000100010000110111001111110000000000000000000000000000000000000
00000000000000000000111100000001110000000100000000000000000000000000000000000000000000000000001110000000001110000100000
00000000000000000000100000000000000000010000000011100000100000110001000000000000000000000001000000000000000000111100000
011110000001110001100100000000000000000000000000000000000001100100000011000111100000111100000011100110000000000000000
1100000000000111111110110000011111000000000110011000000000000000000000110000000000001111111111110000011110000000010001
1000000000000000000000000010100000000000001111111110000001111100001100000011000000000000000000000010000000000000000011111
1111100000011111000011000100110000000000000000000000000000000000010000000000000111111110000000111111000001011001100000 0
00000000001000000000000000011111110000000011111110001001110011100000000000000110000000000000011111100000000000001111110
0111111001110000000000000000000000000000000001000000000000001110000000000011111111111100111000000000000000000100000000
000000000111110000000000111111011101111000000000000000000000001000000000000000011111100000000000011111111100111100000000
0000000000000000000100000000000000000111100000000000001111111011111000000000000000000000001000000000000000100000010000
000011100111111101111000000000000000000000000100000000000000000000000000000111110001111101001100000000000000000000001
0000000000000000000000100000100001111000111001111010000000000010000000000000000000000000000000001111000111001 1
110100000000000000000000000000001000000000000010110000000000000111011000111001000000000000000000000000001000000010 01000
000000111000000000111110010110010000000000000000000001000000000000000000011100000000000001100000001101000011001000000000
000000001100000000000000011110000000001100000001100000001100000001001000000001000000000000011110000000011111000111 00000
00110000000100100000000000000000000001000000000000001110000101111111101111100000110000000001000000000000000000010000000
00000001001101111111111100000110000100001000000000000000000001100000000000000000010110011111111110000011000010000100 00
0000000000000000000100000000000000000111011111111111111110000011000010000100000000000000000001000000000000000110111111111
11111111001011000011000100000000000000000000000000000000000000000110000000111011111111111100001100001100010000000000001
0000000000000100000000111011101111111100011100001100010000000000001001100000000000000000000000001111111000111111001100011 0
00000000000000000000000000000000000000000000001111000000111111000010000100000000000000000000000000010000000000000000000
001110000000000111110000000000000011000000000000000000000000000000000000000000000000100000000000111110000000001100000000 0
000000000000000000000000000000000100000000000111111001001000000110000000000000000000000000000000000000000000000000111101
00110100000011000000000000000000000000000000000000000000000000001000000010011000010000000000000000000000000000000000000
0000000000000000000000000000000000000100000011000000000000000000000000000000000000000000000000000000000000000000110000 00
000000000000000000000000000000000000000000000000000000000000000001000000000000000000000000000000000000000000000000000
000000000000000000001000000100000000000000000000000000000000000000000000000000000000000000001100000000000000001110000000
00000000000000000000000000000000000000000000000010000000000000000001111000000100000000000000000000000000000000000000000
00100000000000000010011111000010000000000000000000000000000000000000000110000000000000000111111110111111000010000000
000000000000000000000000000000100000000000000001111111011111110111111000001000000000000100000000000000000000000000100000
11111100111111111111111110000000000001110000000000000001001000000000000001111101111111100000001101110000000000111000000
000000000001000000000000000011111001111111100000000011110000000000011110000000000000100000000000000011111111001111111 00
000000011111000000001111000000000000000000000000000000011011110011000011111111111111111110011111000000000000000010 00000
000000000001001111111111000111111111111111111111100000000000000000000000000000000011110111111111111001110011111111111111
11000000000000000000000000000000000001111111111110000110111110101111111111111110000000000100000000000000000001111111100
111100000111111111100011111111110000000000000000001100000100000010011110001111111110111011111000000111110000000000001 00
00010000000000000000011111111111110001101101100111100001111111000000000000000000000000000110111011110000111100111001101
11111111111111110000000000110000000000000000011011101111000001111111111110110011111111100010000000001100000000000000000 0
010011101111100001111011111111100001111001110001100000001000000000000000000000000000000000

4. Used Python codes

Code 1 - Converting the binary image into binary code

The program allows users to upload an image through a file dialog and specify its grid dimensions (rows and columns). It processes the image by dividing it into equal cells, analyzes each cell's brightness, and encodes dark areas as binary 1s and light areas as binary 0s.

```
import tkinter as tk
from tkinter import filedialog, simpledialog
from PIL import Image
def analyze_qr_to_binary():
    # Create a hidden root window for dialogs
    root = tk.Tk()
    root.withdraw()
    # Ask user for QR image file
    file_path = filedialog.askopenfilename(
        title="Select QR Code Image",
        filetypes=[("Image Files", "*.png;*.jpg;*.jpeg;*.bmp;*.gif")]
    )
    if not file_path:
        print("No file selected.")
        return
    # Ask for number of columns and rows
    cols = simpledialog.askinteger("Columns", "Enter number of columns:")
    rows = simpledialog.askinteger("Rows", "Enter number of rows:")
    if not cols or not rows:
        print("Invalid input for grid size.")
        return
    # Open the image
    img = Image.open(file_path).convert("L")  # convert to grayscale
    width, height = img.size
    # Compute cell size
    cell_w = width / cols
    cell_h = height / rows
    binary_code = ""
    # Analyze each cell
    for r in range(rows):
        for c in range(cols):
            # Compute cell region
            left = int(c * cell_w)
            top = int(r * cell_h)
            right = int((c + 1) * cell_w)
            bottom = int((r + 1) * cell_h)
            # Crop and compute average brightness
            cell = img.crop((left, top, right, bottom))
            avg_brightness = sum(cell.getdata()) / (cell_w * cell_h)
            # Threshold: darker = 1, lighter = 0
            bit = '1' if avg_brightness < 128 else '0'
            binary_code += bit
    # Output result
    print("\nBinary code representation:\n")
    print(binary_code)
    print("\nDone! Total bits:", len(binary_code))
if __name__ == "__main__":
    analyze_qr_to_binary()
```

Code 2 – compound distribution map

The following source code encodes the compound distribution maps. There are two inputs to be entered: the binary code to be saved and the number of compounds used for encryption. The result is the distribution of each compound over the storing 96-well plate.

```
import tkinter as tk
from tkinter import ttk
from tkinter import messagebox
def divide_binary_string(mother_string, num_compounds):
    daughter_strings = []
    for i in range(1, num_compounds + 1):
        daughter = mother_string[i - 1::num_compounds]
        daughter_strings.append(daughter)
    return daughter_strings
def create_binary_grid(daughter_strings):
    binary_grids = []
    max_length = max(len(daughter) for daughter in daughter_strings)
    padded_daughters = [daughter.ljust(max_length, '0') for daughter in daughter_strings]
    for padded_daughter in padded_daughters:
        grid = [padded_daughter[j:j + 12] for j in range(0, len(padded_daughter), 12)]
        binary_grids.append(grid)
    return binary_grids
def paste_text(input_field, event=None):
    clipboard_content = root.clipboard_get()
    input_field.insert("insert", clipboard_content)
def open_binary_grids(event=None):
    mother_string = binary_entry.get()
    num_compounds = int(num_compounds_entry.get())
    daughter_strings = divide_binary_string(mother_string, num_compounds)
    binary_grids = create_binary_grid(daughter_strings)
    grid_window = tk.Toplevel(root)
    grid_window.title("Binary Grids")
    canvas_widget = tk.Canvas(grid_window)
    canvas_container = tk.Frame(canvas_widget)
    canvas_widget.create_window((0, 0), window=canvas_container, anchor='nw')
    total_width = num_compounds * (12 * 20 + 2 * padding_x)
    total_height = (len(binary_grids[0]) + len(binary_grids[0]) // 8) * 20 + 2 * padding_y
    extra_padding_bottom = 50
    for i, grid in enumerate(binary_grids):
        title_label = tk.Label(canvas_container, text=titles[i], padx=10)
        title_label.grid(row=0, column=i)
        if i == len(binary_grids) - 1:
            total_height += extra_padding_bottom
        canvas_grid = tk.Canvas(canvas_container, width=12 * 20 + 2 * padding_x,
                        height=(len(grid) + len(grid) // 8) * 20 + 2 * padding_y)
        line_names = [chr(97 + (i % num_compounds)) for i in range(len(grid))]
        for row_index, row in enumerate(grid):
            for col_index, cell in enumerate(row):
                x1 = col_index * 20 + padding_x
                y1 = (row_index + row_index // 8) * 20 + padding_y
                x2 = x1 + 20
                y2 = y1 + 20
                if col_index == 0:
                    line_name = line_names[row_index]
                    canvas_grid.create_text(x1 - 2, (y1 + y2) / 2, text=line_name, anchor="e",
                                font=("Arial", 8, "bold"), fill="black")
                if cell == '1':
                    canvas_grid.create_oval(x1, y1, x2, y2, fill='red')
                    column_number = col_index + 1
                    canvas_grid.create_text(x1 + 10, (y1 + y2) / 2, text=str(column_number), anchor="center",
```

```
                                        font=("Arial", 8))
                            else:
                                canvas_grid.create_oval(x1, y1, x2, y2, outline='red')
                    canvas_grid.grid(row=1, column=i)
                    if i == len(binary_grids) - 1:
                        extra_padding = 50
                        canvas_grid.config(width=12 * 20 + 2 * padding_x + extra_padding)
                        total_width += extra_padding
    hbar = tk.Scrollbar(grid_window, orient=tk.HORIZONTAL)
    hbar.pack(side=tk.BOTTOM, fill=tk.X)
    hbar.config(command=canvas_widget.xview)
    canvas_widget.config(xscrollcommand=hbar.set)
    vbar = tk.Scrollbar(grid_window, orient=tk.VERTICAL)
    vbar.pack(side=tk.RIGHT, fill=tk.Y)
    vbar.config(command=canvas_widget.yview)
    canvas_widget.config(yscrollcommand=vbar.set)
    canvas_widget.config(scrollregion=(0, 0, total_width, total_height))
    canvas_widget.bind_all("<MouseWheel>", lambda event: canvas_widget.yview_scroll(int(-1 * (event.delta / 120)), "units"))
    canvas_widget.pack(fill=tk.BOTH, expand=True)
def display_daughter_strings():
    mother_string = binary_entry.get()
    num_compounds = int(num_compounds_entry.get())
    daughter_strings = divide_binary_string(mother_string, num_compounds)
    daughter_window = tk.Toplevel(root)
    daughter_window.title("Daughter Strings")
    text_widget = tk.Text(daughter_window)
    formatted_daughter_strings = "\n\n".join(daughter_strings)
    text_widget.insert("1.0", formatted_daughter_strings)
    text_widget.pack(fill=tk.BOTH, expand=True)
root = tk.Tk()
root.title("Binary Grids and Daughter Strings")
binary_label = tk.Label(root, text="Enter the binary mother string:")
binary_label.pack()
binary_entry = tk.Entry(root)
binary_entry.pack()
num_compounds_label = tk.Label(root, text="Enter the number of compounds:")
num_compounds_label.pack()
num_compounds_entry = tk.Entry(root)
num_compounds_entry.pack()
context_menu = tk.Menu(root, tearoff=0)
context_menu.add_command(label="Paste", command=lambda: paste_text(binary_entry))
binary_entry.bind("<Button-3>", lambda event: context_menu.post(event.x_root, event.y_root))
grid_button = tk.Button(root, text="Open Binary Grids", command=open_binary_grids)
grid_button.pack()
daughter_button = tk.Button(root, text="Display Daughter Strings", command=display_daughter_strings)
daughter_button.pack()
titles = ["Compound I", "Compound II", "Compound III", "Compound IV", "Compound V", "Compound VI",
          "Compound VII", "Compound VIII", "Compound IX", "Compound X", "Compound XI", "Compound XII"]
padding_x = 10
padding_y = 10
root.bind("<Return>", open_binary_grids)
root.bind("<KP_Enter>", open_binary_grids)
root.mainloop()
```

Code 3 – Software for readout

```
import tkinter as tk
from tkinter import filedialog
from PIL import Image, ImageTk
import numpy as np
class ImageViewer:
    def __init__(self, root):
        self.root = root
        self.root.title("Image Viewer")
        # Canvas for image
        self.canvas = tk.Canvas(root, cursor="cross")
        self.canvas.pack(fill=tk.BOTH, expand=True)
        # Frame for buttons
        button_frame = tk.Frame(root)
        button_frame.pack(fill=tk.X)
        self.button_open = tk.Button(button_frame, text="Open Image", command=self.open_image)
        self.button_open.grid(row=0, column=0, padx=5, pady=5)
        self.button_scan = tk.Button(button_frame, text="Automated Scan", command=self.automated_scan)
        self.button_scan.grid(row=0, column=1, padx=5, pady=5)
        self.button_v_values = tk.Button(button_frame, text="V Values", command=self.calculate_and_display_v_values)
        self.button_v_values.grid(row=0, column=2, padx=5, pady=5)
        self.button_print_squares = tk.Button(button_frame, text="Print 1/0 in Squares", command=self.print_ones_and_zeros)
        self.button_print_squares.grid(row=0, column=3, padx=5, pady=5)
        self.button_print_distances = tk.Button(button_frame, text="Print Relative Distances", command=self.print_relative_distances)
        self.button_print_distances.grid(row=0, column=4, padx=5, pady=5)
        self.button_generate_binary_string = tk.Button(button_frame, text="Generate Binary String",
command=self.generate_and_print_binary_string)
        self.button_generate_binary_string.grid(row=0, column=5, padx=5, pady=5)
        self.button_show_binary_string_window = tk.Button(button_frame, text="Show Binary String Window",
command=self.open_binary_string_window)
        self.button_show_binary_string_window.grid(row=0, column=6, padx=5, pady=5)
        self.canvas.bind("<Double-1>", self.set_point)
        self.canvas.bind("<ButtonPress-1>", self.on_press)
        self.canvas.bind("<B1-Motion>", self.on_drag)
        self.canvas.bind("<ButtonRelease-1>", self.on_release)
        self.canvas.bind("<Motion>", self.display_hover_info)
        self.canvas.bind("<Button-2>", self.toggle_square)  # Middle mouse button click
        self.horizontal_relative_distances = [
            0.0, 0.07526881720430108, 0.15591397849462366, 0.21774193548387097,
            0.3279569892473118, 0.4435483870967742, 0.5833333333333334,
            0.6935483870967742, 0.7768817204301075, 0.9516129032258065, 1.0
        ]
        self.vertical_relative_distances = [
            0.0, 0.09018036072144289, 0.1683366733466934, 0.2545090180360721,
            0.3346693386773547, 0.4168336673346693, 0.503006012024048,
            0.5851703406813628, 0.6713426853707415, 0.7595190380761523,
            0.8356713426853707, 0.9198396793587175, 1.0
        ]
        self.point1 = None
        self.point2 = None
        self.point_set = False
        self.horizontal_lines = {}
        self.vertical_lines = {}
        self.bands = {}
        self.values = {}
        self.saved_values = {}
        self.drag_data = {}
        self.hover_label = tk.Label(root, text='Hover over the image to see V values')
        self.hover_label.pack()
        self.hover_horizontal_line = None
        self.hover_vertical_line = None
        # Initialize dictionary to store binary values
        self.bands_binary_values = {}
    def open_image(self):
        self.reset()
```

```
    file_path = filedialog.askopenfilename(filetypes=[("Image files", "*.jpg;*.jpeg;*.png;*.bmp;*.gif")])
    if file_path:
        try:
            self.img = Image.open(file_path)
            self.img.thumbnail((800, 800))
            self.img_tk = ImageTk.PhotoImage(self.img)
            self.canvas_img = self.canvas.create_image(0, 0, anchor=tk.NW, image=self.img_tk)
            self.canvas.config(scrollregion=self.canvas.bbox(tk.ALL))
            img_width, img_height = self.img.size
            self.canvas.config(width=img_width, height=img_height)
            self.root.geometry(f"{img_width}x{img_height + 50}")
            self.root.title(f"Image Viewer - {file_path.split('/')[-1]}")
        except Exception as e:
            print(f"Failed to load image: {e}")
def reset(self):
    self.canvas.delete("grid")
    self.canvas.delete("hover_line")
    self.canvas.delete("band")
    self.canvas.delete("v_value")
    self.horizontal_lines.clear()
    self.vertical_lines.clear()
    self.bands.clear()
    self.values.clear()
    self.saved_values.clear()
    self.point1 = None
    self.point2 = None
    self.point_set = False
    if self.hover_horizontal_line:
        self.canvas.delete(self.hover_horizontal_line)
        self.hover_horizontal_line = None
    if self.hover_vertical_line:
        self.canvas.delete(self.hover_vertical_line)
        self.hover_vertical_line = None
    self.hover_label.config(text='Hover over the image to see V values')
def set_point(self, event):
    if not self.point_set:
        self.point1 = (event.x, event.y)
        self.point_set = True
        print(f"Point 1 set at: {self.point1}")
    else:
        self.point2 = (event.x, event.y)
        self.point_set = False
        print(f"Point 2 set at: {self.point2}")
        self.draw_grid()
def draw_grid(self):
    if not self.point1 or not self.point2:
        return
    for rel_y in self.horizontal_relative_distances:
        y = self.point1[1] + rel_y * (self.point2[1] - self.point1[1])
        line_id = self.canvas.create_line(0, y, self.canvas.winfo_width(), y, fill="blue", tags="grid")
        self.horizontal_lines[line_id] = y
    for rel_x in self.vertical_relative_distances:
        x = self.point1[0] + rel_x * (self.point2[0] - self.point1[0])
        line_id = self.canvas.create_line(x, 0, x, self.canvas.winfo_height(), fill="red", tags="grid")
        self.vertical_lines[line_id] = x
def on_press(self, event):
    x, y = event.x, event.y
    for line_id, line_y in self.horizontal_lines.items():
        if abs(y - line_y) < 5:
            self.drag_data = {"item": line_id, "type": "horizontal", "start_y": y}
            break
    for line_id, line_x in self.vertical_lines.items():
        if abs(x - line_x) < 5:
            self.drag_data = {"item": line_id, "type": "vertical", "start_x": x}
            break
def on_drag(self, event):
```

```
    if "item" in self.drag_data:
        item_type = self.drag_data["type"]
        item_id = self.drag_data["item"]
        if item_type == "horizontal":
            new_y = event.y
            self.canvas.coords(item_id, 0, new_y, self.canvas.winfo_width(), new_y)
            self.horizontal_lines[item_id] = new_y
        elif item_type == "vertical":
            new_x = event.x
            self.canvas.coords(item_id, new_x, 0, new_x, self.canvas.winfo_height())
            self.vertical_lines[item_id] = new_x
        self.update_bands()
def on_release(self, event):
    self.drag_data = {}
def update_bands(self):
    self.canvas.delete("band")
    self.bands.clear()
    img_width, img_height = self.img.size
    band_width = img_width / 40  # Updated to 1/40
    band_height = img_height / 40  # Updated to 1/40
    horizontal_lines_sorted = sorted(self.horizontal_lines.values())
    vertical_lines_sorted = sorted(self.vertical_lines.values())
    for h_line_y in horizontal_lines_sorted:
        for v_line_x in vertical_lines_sorted:
            self.canvas.create_rectangle(v_line_x - band_width / 2, h_line_y - band_height / 2,
                                         v_line_x + band_width / 2, h_line_y + band_height / 2,
                                         outline='#D3D3D3', tags="band")  # Lighter gray color
            self.bands[(v_line_x, h_line_y)] = (band_width, band_height)
            # Initialize binary value to 0
            self.bands_binary_values[(v_line_x, h_line_y)] = 0
def convert_to_bands(self):
    if not hasattr(self, 'img'):
        print("No image loaded.")
        return
    self.canvas.delete("band")
    self.bands.clear()
    img_width, img_height = self.img.size
    band_width = img_width / 40  # Updated to 1/40
    band_height = img_height / 40  # Updated to 1/40
    horizontal_lines_sorted = sorted(self.horizontal_lines.values())
    vertical_lines_sorted = sorted(self.vertical_lines.values())
    for h_line_y in horizontal_lines_sorted:
        for v_line_x in vertical_lines_sorted:
            if v_line_x is not None:
                self.canvas.create_rectangle(v_line_x - band_width / 2, h_line_y - band_height / 2,
                                             v_line_x + band_width / 2, h_line_y + band_height / 2,
                                             outline='#D3D3D3', tags="band")  # Lighter gray color
                self.bands[(v_line_x, h_line_y)] = (band_width, band_height)
                # Initialize binary value to 0
                self.bands_binary_values[(v_line_x, h_line_y)] = 0
def print_relative_distances(self):
    for line_id, line_y in self.horizontal_lines.items():
        relative_distance = line_y / self.canvas.winfo_height()
        print(f"Relative distance for horizontal line {line_id}: {relative_distance:.4f}")
    for line_id, line_x in self.vertical_lines.items():
        relative_distance = line_x / self.canvas.winfo_width()
        print(f"Relative distance for vertical line {line_id}: {relative_distance:.4f}")
def automated_scan(self):
    if not hasattr(self, 'img'):
        print("No image loaded.")
        return
    img_width, img_height = self.img.size
    scanner = AutomatedScanning(self.img, self.canvas, self.horizontal_lines, self.vertical_lines)
    for line_id in self.horizontal_lines.keys():
        new_position = scanner.move_line_to_lowest_v(line_id, img_height, axis="horizontal")
        print(f"Moved horizontal line {line_id} to new position: {new_position}")
```

```
        for line_id in self.vertical_lines.keys():
            new_position = scanner.move_line_to_lowest_v(line_id, img_width, axis="vertical")
            print(f"Moved vertical line {line_id} to new position: {new_position}")
        self.convert_to_bands()
    def display_hover_info(self, event):
        if not hasattr(self, 'img'):
            return
        x, y = event.x, event.y
        if 0 <= x < self.img.width and 0 <= y < self.img.height:
            img_hsv = self.img.convert('HSV')
            img_np = np.array(img_hsv)
            v_value = img_np[y, x, 2]
            self.hover_label.config(text=f'Hover position: ({x}, {y}), V: {v_value}')
            if self.hover_horizontal_line:
                self.canvas.coords(self.hover_horizontal_line, 0, y, self.canvas.winfo_width(), y)
            else:
                self.hover_horizontal_line = self.canvas.create_line(0, y, self.canvas.winfo_width(), y,
                                                                    fill="yellow", tags="hover_line")
            if self.hover_vertical_line:
                self.canvas.coords(self.hover_vertical_line, x, 0, x, self.canvas.winfo_height())
            else:
                self.hover_vertical_line = self.canvas.create_line(x, 0, x, self.canvas.winfo_height(),
                                                                  fill="yellow", tags="hover_line")
    def calculate_and_display_v_values(self):
        if not hasattr(self, 'img'):
            print("No image loaded.")
            return
        # Remove existing lines and text
        self.canvas.delete("grid")  # Assuming all lines are tagged as "grid"
        self.canvas.delete("v_value")  # Remove text tags
        # Remove existing bands to avoid duplication
        self.canvas.delete("band")
        self.bands_binary_values = {}  # Dictionary to store binary values for each band
        img_hsv = self.img.convert('HSV')
        img_np = np.array(img_hsv)
        # Calculate and display V values for each rectangle
        for (v_line_x, h_line_y), (band_width, band_height) in self.bands.items():
            rect_x1 = int(v_line_x - band_width / 2)
            rect_y1 = int(h_line_y - band_height / 2)
            rect_x2 = int(v_line_x + band_width / 2)
            rect_y2 = int(h_line_y + band_height / 2)
            # Extract the V channel values for the rectangle
            rect_v_values = img_np[rect_y1:rect_y2, rect_x1:rect_x2, 2]  # Extract V channel values
            avg_v = int(np.mean(rect_v_values))  # Convert to int for no decimals
            # Calculate and display average V values for adjacent segments
            left_x = v_line_x - band_width
            right_x = v_line_x + band_width
            # Check if segments are within image boundaries
            avg_v_left = self.calculate_average_v_for_segment(h_line_y, left_x) if left_x >= 0 else None
            avg_v_right = self.calculate_average_v_for_segment(h_line_y, right_x) if right_x < img_np.shape[1] else None
            # Handle the case where only one adjacent segment is available
            if avg_v_left is not None and avg_v_right is not None:
                avg_adjacent_v = int((avg_v_left + avg_v_right) / 2)
            elif avg_v_left is not None:
                avg_adjacent_v = avg_v_left
            elif avg_v_right is not None:
                avg_adjacent_v = avg_v_right
            else:
                avg_adjacent_v = 0  # Or handle this case differently if needed
            # Determine binary value and store it
            if avg_adjacent_v != 0:  # Avoid division by zero
                ratio = avg_v / avg_adjacent_v
                binary_value = 1 if ratio < 0.9 else 0
            else:
                binary_value = 0  # Default value if adjacent V is zero
            self.bands_binary_values[(v_line_x, h_line_y)] = binary_value
```

```
        # Draw the rectangle with thin borders based on the binary value
        color = 'green' if binary_value == 1 else 'red'
        self.canvas.create_rectangle(v_line_x - band_width / 2, h_line_y - band_height / 2,
                                     v_line_x + band_width / 2, h_line_y + band_height / 2,
                                     outline=color, width=1, tags="band")
def calculate_average_v_for_segment(self, line_y, segment_x):
    """
    Helper function to calculate the average V value for a given vertical segment.
    """
    if not hasattr(self, 'img'):
        print("No image loaded.")
        return 0
    img_hsv = self.img.convert('HSV')
    img_np = np.array(img_hsv)
    # Define segment boundaries
    x = int(segment_x)
    segment_width = img_np.shape[1] // 40  # Adjust based on desired segment width
    # Ensure that we are within the image boundaries
    if x - segment_width // 2 < 0:
        segment_x1 = 0
    else:
        segment_x1 = x - segment_width // 2
    if x + segment_width // 2 >= img_np.shape[1]:
        segment_x2 = img_np.shape[1]
    else:
        segment_x2 = x + segment_width // 2
    # Extract the V channel values for the segment
    segment_v_values = img_np[line_y - 10:line_y + 10, segment_x1:segment_x2,
                2]  # Adjust height based on segment height
    return int(np.mean(segment_v_values))
def toggle_square(self, event):
    if not hasattr(self, 'img'):
        return
    x, y = event.x, event.y
    band_width = self.img.width / 40  # Band width used in `update_bands`
    band_height = self.img.height / 40  # Band height used in `update_bands`
    # Find the rectangle in which the middle mouse button was clicked
    for (v_line_x, h_line_y), (width, height) in self.bands.items():
        rect_x1 = int(v_line_x - width / 2)
        rect_y1 = int(h_line_y - height / 2)
        rect_x2 = int(v_line_x + width / 2)
        rect_y2 = int(h_line_y + height / 2)
        if rect_x1 <= x <= rect_x2 and rect_y1 <= y <= rect_y2:
            # Toggle the binary value
            current_value = self.bands_binary_values.get((v_line_x, h_line_y), 0)
            new_value = 1 - current_value
            self.bands_binary_values[(v_line_x, h_line_y)] = new_value
            # Update the color of the rectangle
            color = 'green' if new_value == 1 else 'red'
            self.canvas.create_rectangle(v_line_x - width / 2, h_line_y - height / 2,
                                         v_line_x + width / 2, h_line_y + height / 2,
                                         outline=color, tags="band")
            break
def print_ones_and_zeros(self):
    # Calculate the width of each band
    band_width = self.img.width / 40  # Adjust this if the number of bands changes
    # Convert keys to list to access the first column
    band_keys = list(self.bands_binary_values.keys())
    if not band_keys:
        return  # Return if there are no bands
    # Determine the x-coordinate of the first column
    first_column_x = band_keys[0][0] - band_width / 2
    # Iterate over each rectangle in the grid
    for (v_line_x, h_line_y), binary_value in self.bands_binary_values.items():
        # Skip printing for the rectangles in the first column
        if v_line_x < first_column_x + band_width:
```

```
                continue  # Skip the first column
            # Determine the text color based on the binary value
            text_color = 'white' if binary_value == 1 else 'black'
            # Draw the text in the center of each square
            self.canvas.create_text(v_line_x, h_line_y, text=str(binary_value),
                                    fill=text_color, font=("Arial", 12, "bold"), tags="band")
    def generate_and_print_binary_string(self):
        if not hasattr(self, 'bands_binary_values') or not self.bands_binary_values:
            print("No binary values available.")
            return ""
        # Convert keys to list to access the first column
        band_keys = list(self.bands_binary_values.keys())
        if not band_keys:
            return ""  # Return an empty string if there are no bands
        # Determine the x-coordinate of the first column
        band_width = self.img.width / 40  # Adjust this if the number of bands changes
        first_column_x = band_keys[0][0] - band_width / 2
        # Sort bands first by horizontal coordinate (column) then by vertical coordinate (row)
        sorted_bands = sorted(self.bands_binary_values.keys(), key=lambda x: (x[0], x[1]))
        # Create binary string, skipping the first column
        binary_string = ''.join(
            str(self.bands_binary_values[band])
            for band in sorted_bands
            if band[0] >= first_column_x + band_width
        )
        print(f"Binary string: {binary_string}")
        return binary_string
    def open_binary_string_window(self):
        binary_string = self.generate_and_print_binary_string()
        # Check if the window already exists
        if hasattr(self, 'binary_string_window') and self.binary_string_window is not None:
            # Append the new binary string to the existing text widget
            self.binary_string_window.text_window.insert(tk.END, "\n" + binary_string)
            return
        # Create a new top-level window
        self.binary_string_window = tk.Toplevel(self.root)
        self.binary_string_window.title("Binary String")
        # Create a Text widget in the new window
        text_window = tk.Text(self.binary_string_window, height=10, width=50)
        text_window.pack(padx=10, pady=10)
        # Insert the binary string into the Text widget
        text_window.insert(tk.END, binary_string)
        # Store a reference to the text widget for future use
        self.binary_string_window.text_window = text_window
class AutomatedScanning:
    def __init__(self, img, canvas, horizontal_lines, vertical_lines):
        self.img = img
        self.canvas = canvas
        self.horizontal_lines = horizontal_lines
        self.vertical_lines = vertical_lines
        self.img_hsv = self.img.convert('HSV')
        self.img_np = np.array(self.img_hsv)
    def move_line_to_lowest_v(self, line_id, img_size, axis, num_scans=5):
        line_pos = self.horizontal_lines.get(line_id, self.vertical_lines.get(line_id))
        max_distance = img_size / 70
        distances = [i * max_distance / (num_scans - 1) for i in range(num_scans)]
        min_avg_v = float('inf')
        best_line_position = line_pos
        if axis == "horizontal":
            for distance in distances:
                for sign in [-1, 1]:
                    scan_y = int(round(line_pos + sign * distance))
                    if 0 <= scan_y < self.img_np.shape[0]:
                        avg_v = np.mean(self.img_np[scan_y, :, 2])
                        if avg_v < min_avg_v:
                            min_avg_v = avg_v
```

```
                    best_line_position = scan_y
            self.canvas.coords(line_id, 0, best_line_position, self.canvas.winfo_width(), best_line_position)
            self.horizontal_lines[line_id] = best_line_position
        else:  # axis == "vertical"
            for distance in distances:
                for sign in [-1, 1]:
                    scan_x = int(round(line_pos + sign * distance))
                    if 0 <= scan_x < self.img_np.shape[1]:
                        avg_v = np.mean(self.img_np[:, scan_x, 2])
                        if avg_v < min_avg_v:
                            min_avg_v = avg_v
                            best_line_position = scan_x
            self.canvas.coords(line_id, best_line_position, 0, best_line_position, self.canvas.winfo_height())
            self.vertical_lines[line_id] = best_line_position
        return best_line_position
if __name__ == "__main__":
    root = tk.Tk()
    viewer = ImageViewer(root)
    root.mainloop()
```

Code 4 – Binary code similarity

```
def calculate_similarity(binary1, binary2):
    min_length = min(len(binary1), len(binary2))
    binary1 = binary1[:min_length]
    binary2 = binary2[:min_length]
    total_places = len(binary1)
    same_places = sum(1 for b1, b2 in zip(binary1, binary2) if b1 == b2)
    similarity = same_places / total_places
    return similarity
def highlight_diff_positions(binary1, binary2):
    highlighted_str1 = binary1
    highlighted_str2 = ""
    for b1, b2 in zip(binary1, binary2):
        if b1 == b2:
            highlighted_str2 += b2
        else:
            highlighted_str2 += "*"
    return highlighted_str1, highlighted_str2
def name_lines():
    line_names = []
    for i in range(1, 3):
        for letter in "abcdefgh":
            for j in range(1, 13):
                line_names.append(f"{i}{letter}{j}")
    return line_names
if __name__ == "__main__":
    binary1 = input("Enter the first binary string: ")
    binary2 = input("Enter the second binary string: ")
    similarity = calculate_similarity(binary1, binary2)
    highlighted_str1, highlighted_str2 = highlight_diff_positions(binary1, binary2)
    line_names = name_lines()
    formatted_binary1 = ' '.join([highlighted_str1[i:i + 8] for i in range(0, len(highlighted_str1), 8)])
    formatted_binary2 = ' '.join([highlighted_str2[i:i + 8] for i in range(0, len(highlighted_str2), 8)])
    # Split the formatted binary strings into individual 8-bit strings
    formatted_binary1 = formatted_binary1.split()
    formatted_binary2 = formatted_binary2.split()
    max_len = max(len(formatted_binary1), len(formatted_binary2))
    formatted_binary1 += [''] * (max_len - len(formatted_binary1))
    formatted_binary2 += [''] * (max_len - len(formatted_binary2))
    print("Line Name\tBinary String 1\tBinary String 2")
    # Iterate through the lines and binary strings, using the minimum length to avoid index out of range
    for i in range(min(len(line_names), len(formatted_binary1))):
        name = line_names[i]
        bin1 = formatted_binary1[i]
        bin2 = formatted_binary2[i]
        # Create a list of error positions within the 8-bit string
        error_positions = [pos for pos, char in enumerate(bin2) if char == '*']
        if error_positions:
            error_messages = []  # Store multiple error messages for this line
            for position in error_positions:
                real_position = position + 1  # Add 1 to get the correct position
                if bin1[position] == '1':
                    error_messages.append(f"Compound {real_position} is missing")
                else:
                    error_messages.append(f"Compound {real_position} should not be contained")
            error_message = ", ".join(error_messages)
            print(f"{name.rjust(8)}\t{bin1.rjust(8)}\t{bin2.rjust(8)} (error: {error_message})")
        else:
            print(f"{name.rjust(8)}\t{bin1.rjust(8)}\t{bin2.rjust(8)}")
    print(f"Similarity: {similarity:.2%}")
```

5. Pictures of the TLC plates

TLC pictures for recovery of QR code

1A

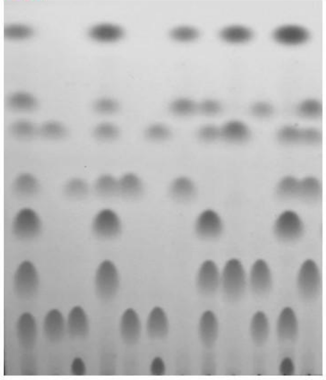

1B

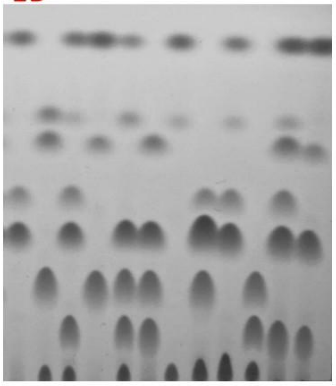

1C

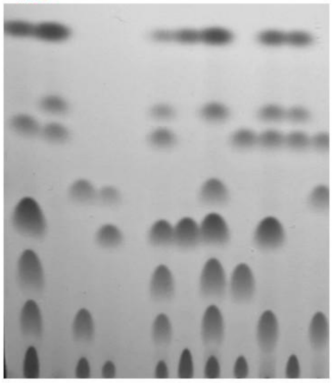

1D

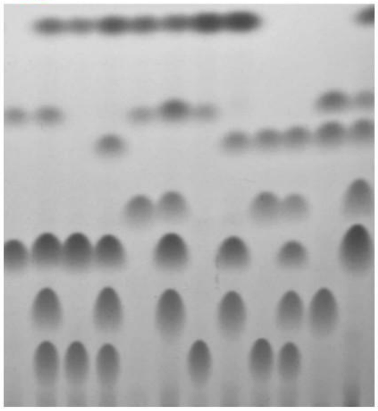

1E

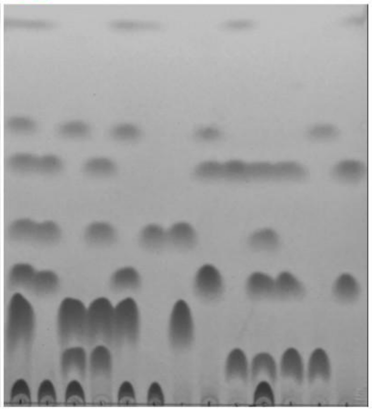

1F

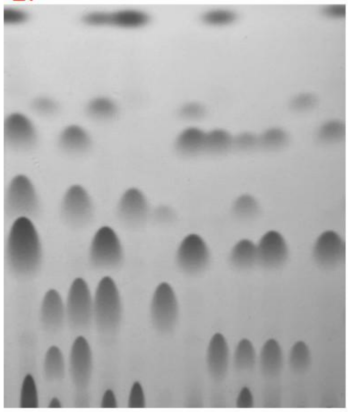

1G

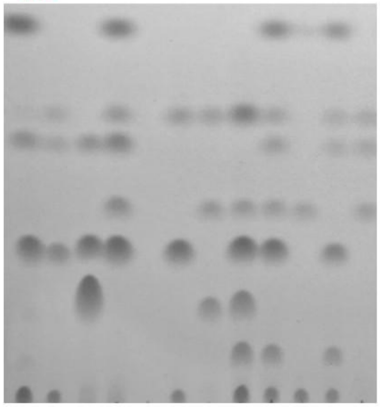

1H

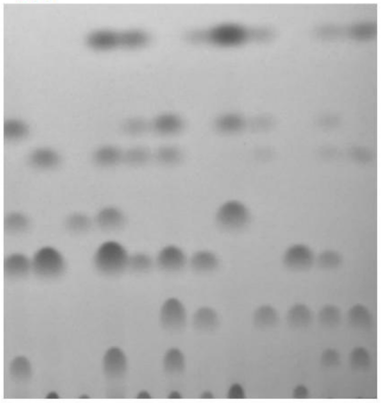

2A

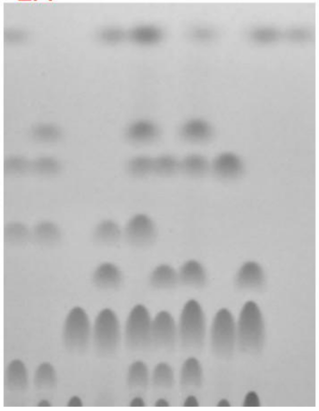

TLC pictures for recovery of Charles IV image

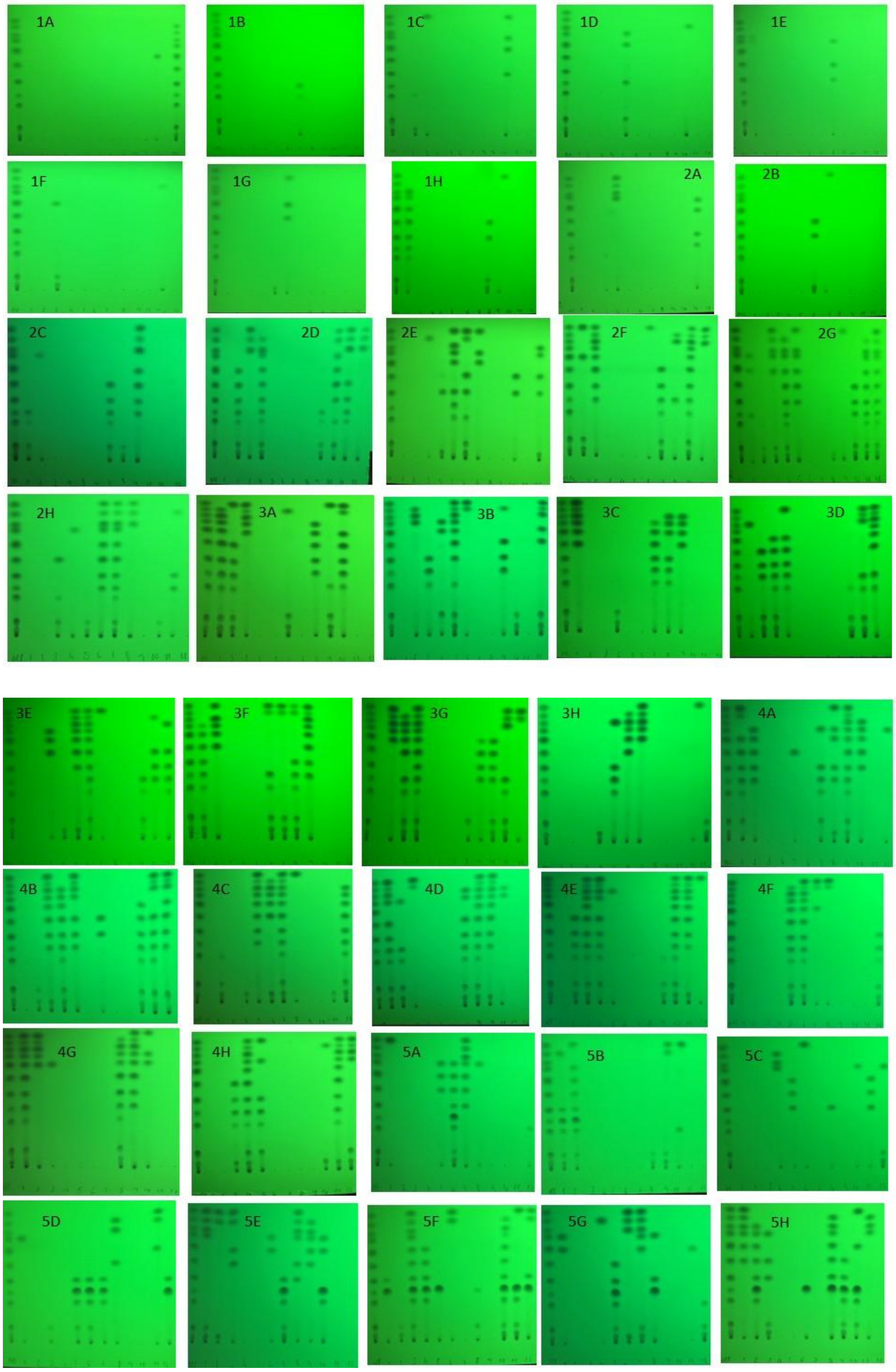

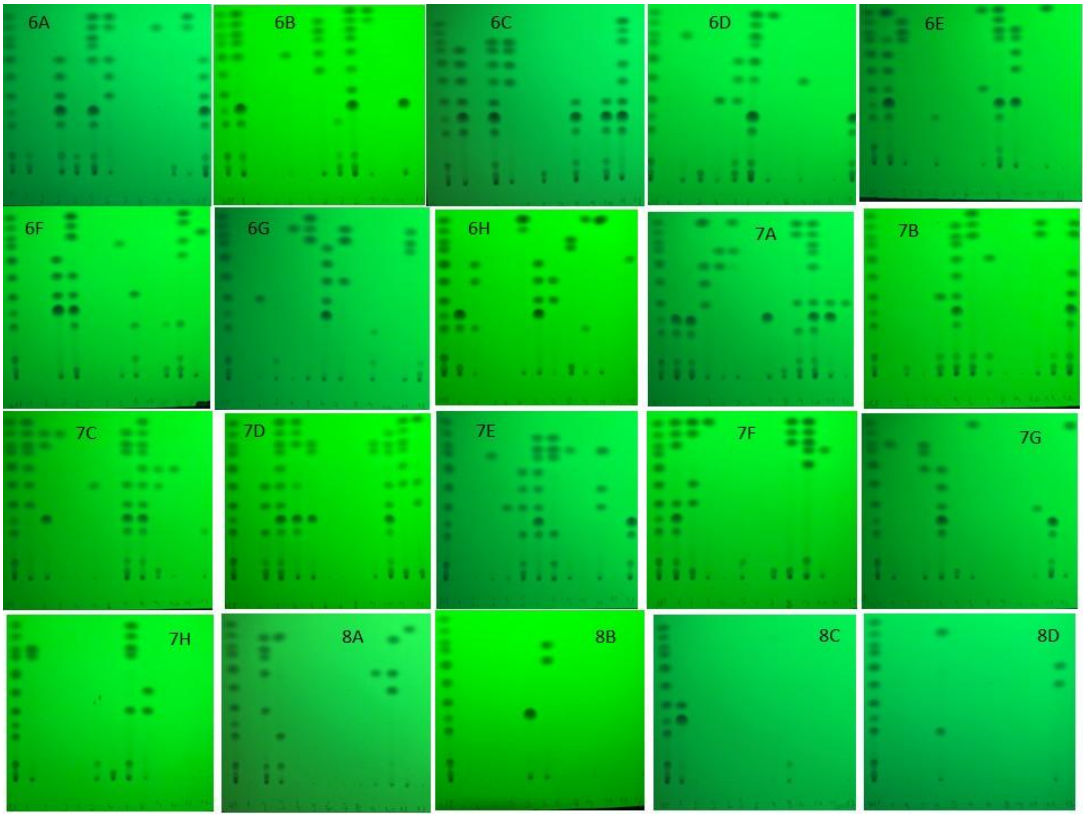
6A
6B
6C
6D
6E
6F
6G
6H
7A
7B
7C
7D
7E
7F
7G
7H
8A
8B
8C
8D

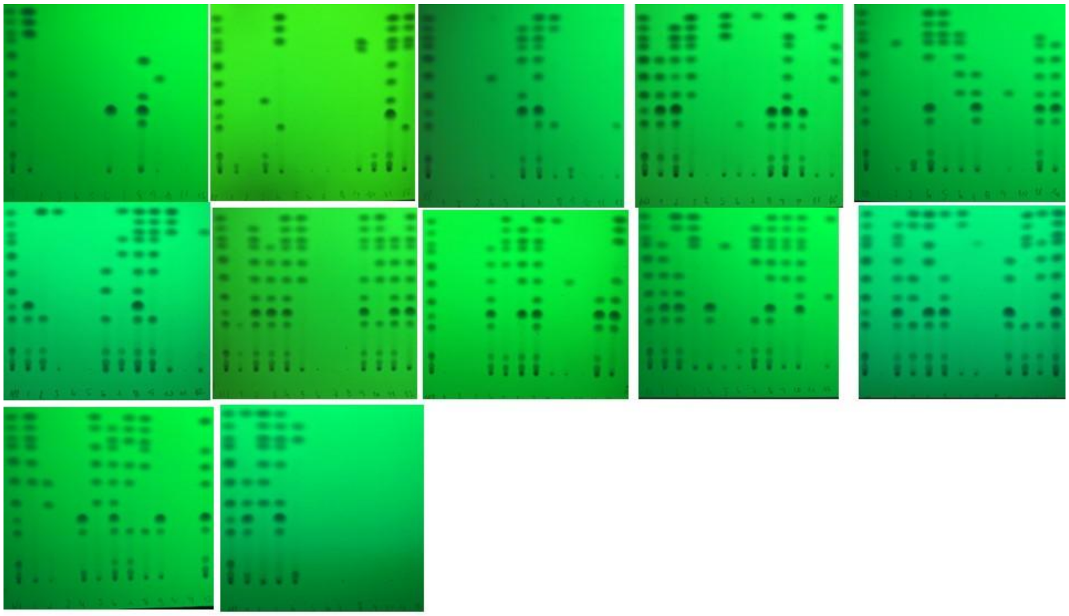

6. References

[1] Shabbir, S., Lee, Y. & Rhee, H. Au(III) catalyst supported on a thermoresponsive hydrogel and its application to the A-3 coupling reaction in water. *J. Catal.* **322**, 104–108 (2015).

[2] Cui, J. *et al.* Silver-Mediated Organic Transformations of Propargylamines to Enones, α-Thioketones, and Isochromans. *ChemistrySelect* **4**, 1476–1482 (2019).

[3] Brambilla, E. *et al.* Silver-catalysed A3-coupling reactions in phenylacetic acid/alkylamine N-oxide eutectic mixture under dielectric heating: An alternative approach to propargylamines. *Appl. Organomet. Chem.* 1–10 (2022) doi:10.1002/aoc.6669.

[4] Mateus, M., Kiss, A., Císařová, I., Karpiński, T. M. & Rycek, L. Synthesis of silver complexes with chelating bidentate N -heterocyclic ligands, their application in catalytic A 3 coupling, and as antimicrobial agents. *Appl. Organomet. Chem.* **37**, 675–687 (2023).

[5] Yi, R. *et al.* Expeditious and highly efficient synthesis of propargylamines using a Pd-Cu nanowires catalyst under solvent-free conditions. *Appl. Organomet. Chem.* **33**, 1–7 (2019).

[6] Sampani, S. I. *et al.* Shedding light on the use of Cu(ii)-salen complexes in the A3 coupling reaction. *Dalt. Trans.* **49**, 289–299 (2020).

[7] Liu, Z., Yuan, D. & Su, Y. A Novel and Versatile Copper-Nanomagnetic Catalyst for Synthesis of Propargylamines and Diaryl Sulfides. *Catal. Letters* **153**, 698–712 (2023).

[8] S. Zhang, Y. Huang, T. Lin, M. Xue, S. Liu, H. Deng & Y. Li. Novel Cu-Based and Pd-Based CMC–CA–PVA Composite Films as Efficient Dip Catalysts for A3 Coupling and Suzuki–Miyaura Coupling Reactions. Cellulose **32**, 1645–1665 (2025).
